# Strengthening the delivery of integrated physical health care for adults experiencing serious mental illness: a scoping review of interventions in mental health settings

**DOI:** 10.3389/frhs.2025.1570100

**Published:** 2025-06-20

**Authors:** Munazzah Ambreen, Sihan Zhang, Osnat C. Melamed, Christopher Canning, Brian Lo, Sri Mahavir Agarwal, Amer M. Burhan, M. Elisabeth Del Giudice, Mary Rose van Kesteren, Barna Konkolÿ Thege, Sanjeev Sockalingam, Terri Rodak, Tania Tajirian, Caroline Walker, Vicky Stergiopoulos

**Affiliations:** ^1^ Centre for Addiction and Mental Health, Toronto, ON, Canada; ^2^ Department of Family and Community Medicine, University of Toronto, Toronto, ON, Canada; ^3^ Waypoint Research Institute, Waypoint Centre for Mental Health Care, Penetanguishene, ON, Canada; ^4^ Department of Psychiatry, University of Toronto, Toronto, ON, Canada; ^5^ Institute of Health Policy, Management and Evaluation, University of Toronto, Toronto, ON, Canada; ^6^ Ontario Shores Centre for Mental Health Sciences, Whitby, ON, Canada

**Keywords:** serious mental illness, integrated care, reverse integration, premature mortality, service delivery

## Abstract

Individuals living with serious mental illness (SMI) face significant barriers to accessing appropriate physical health care, poorer associated health outcomes and premature mortality compared to the general population. This scoping review examines service delivery models and clinical practices supporting the integration of physical health care for adults with SMI within mental health settings, and their outcomes. Searches of four academic databases yielded 65 academic articles. Most integrated service delivery models were implemented in community mental health settings in the United States and incorporated elements of Wagner's Chronic Care Model, emphasizing delivery-system redesign, patient self-management support and use of clinical information systems. In most outcome studies, integrated care models were associated with improvements in primary care access and preventative screening rates, while other physical health indicators and emergency and inpatient service use demonstrated promising but mixed results. Implementation challenges of integrated service delivery models included securing financial resources and maintaining effective use of clinical information systems, among others. Successful implementation was facilitated by effective teamwork, care coordination, and administrative and leadership support. Study findings highlight the complexity of integrating physical health care in mental health settings, and the longer timeframes needed to observe changes in some outcomes. The review further underscores the need for ongoing efforts to advance integrated care delivery in mental health settings and the importance of longitudinal data collection to fully assess and optimize the implementation and outcomes of these interventions.

**Systematic Review Registration:** https://doi.org/10.17605/OSF.IO/3T9VK.

## Introduction

Serious mental illnesses (SMIs), such as schizophrenia, bipolar disorder and treatment resistant depression, are chronic health conditions that severely impact the everyday functioning and quality of life of affected individuals (1–7). Adults with SMI between 18 and 49 years of age are 3.2 and 2.5 times more likely to die from cardiovascular disease (CVD) and stroke respectively, and die on average 10–20 years earlier compared to the general population (8, 9). Although findings on the relationship between SMI and cancer mortality remain mixed, adults with SMI 50–75 years of age were 1.32 more likely to die of respiratory cancer compared to the general population in one study (9), while lower cancer screening rates among adults with SMI are well documented (10). Multiple factors contribute to premature mortality among adults living with SMI, including individual level, health system level, and social and community level factors (11–13). Illness-related and behavior specific factors can hinder individuals’ ability to manage their physical health and adhere to treatment regiments (14–18). Additionally, antipsychotic medications commonly used to treat SMI are associated with significant side effects, such as weight gain and metabolic imbalances (19, 20). Furthermore, limited access to comprehensive primary care, poor service design and engagement, and diagnostic overshadowing make screening and timely treatment of common health conditions challenging for this population (3, 21–23). Collectively these barriers result in poor quality and experiences of care and growing health disparities among adults with SMI, further accentuated by poverty, homelessness and social isolation (24–30).

To date, most efforts to integrate physical and mental health service delivery have focused on introducing mental health professionals into primary care settings to address the needs of individuals with mild and moderate mental health conditions (31–34). Generally, these models have demonstrated improved patient outcomes and adaptability to the needs of diverse subpopulations, with implementation costs typically offset by longer term healthcare expenditures (35, 36). Less is known about how best to deliver integrated physical and mental health care within mental health settings, such as psychiatric hospitals and community mental health teams serving adults with SMI, who are less likely to engage in primary care services (33, 37). Recent literature has conceptualized such efforts as “reverse integration”, a term referring to providing collaborative physical and mental health care within behavioral rather than primary care settings (38–40).

Various reverse integration models have been described in the international literature in recent years, along with policy development and targeted initiatives in some countries (38, 39, 41–43). Furthermore, research efforts have examined the impact of peer-led self-management, provider education, electronic reminders, and other patient-centered approaches to promote attention to and treatment of chronic health conditions in this population (44–53). Finally, in Canada and other countries, without specific policy or practice mandates, mental health service organizations have been increasingly introducing hospitalist physicians or nurse practitioners to support the physical health needs of adults with SMI in their setting (54–57). Yet despite growing awareness of the mortality gap and efforts to address these health disparities, in most jurisdictions there is no actionable roadmap to advance physical and mental health care integration and delivery within mental health services, the “health home” of this population (4, 58–61).

To help inform service redesign efforts, we undertook a scoping review of the literature to understand the extent and type of evidence in relation to service delivery models and clinical practices that are used to support the integration and delivery of physical health care to individuals with SMI within mental health settings. Two research questions were addressed: [1] What service delivery models and clinical practices are used to support the delivery of physical health care to individuals with serious mental illness in mental health settings?; and [2] What are the outcomes of these models and practices?

## Methods

To effectively capture the extensive scope and depth of this field, we undertook a scoping review of the academic literature. The methodology for this review was based on the framework developed by Arksey and O'Malley (62) and adhered to the reporting guidelines of the Preferred Reporting Items for Systematic reviews and Meta-Analyses (PRISMA) extension for scoping reviews (63). The scoping review was registered via OSF (https://doi.org/10.17605/OSF.IO/3T9VK). Notably, the study team included clinicians, health service researchers, persons with lived experience of SMI and family members, who jointly framed the research questions, interpreted findings, and co-authored the manuscript.

### Inclusion and exclusion criteria

The Substance Abuse and Mental Health Services Administration in the U.S. defines SMI as a diagnosable mental, behavioral, or emotional disorder that substantially interferes with a person's life and ability to function (7). Articles were eligible for this scoping review if: [1] the population of interest included individuals over the age of 18 living with SMI, such as bipolar disorder, treatment refractory depression or schizophrenia, with no other demographic constraints; the age cutoff of 18 was selected to focus on the adult population, as mental health needs and treatment approaches can differ between adults and minors (64); [2] articles focused on the concept of reverse integration, defined as the provision of physical health care services within mental health settings to address physical health needs and prevent physical health decline (40, 65); [3] they were published as peer-reviewed academic journal articles and book chapters; [4] they were published between January 1, 2010 and June 6, 2024, to capture the most recent studies and reflect evolving practices and developments in the field over the past decade; and [5] they were written in English language. There were no methodological restrictions on article eligibility.

Articles were excluded if they: [1] focused on individuals without SMI (e.g., anxiety); [2] did not discuss the provision of physical health services (e.g., focused on psychosocial care); [3] targeted a single health dimension (e.g., smoking, obesity, physical fitness, metabolic health, sexual health, oral health, sleep), lifestyle modifications, or self-management skills training without attention to physical health needs comprehensively; [4] were not or not clearly stated to be set in mental health settings [5] did not describe or evaluate service delivery models or clinical practices; [6] were conference abstracts, dissertations, theses, reviews or study protocols. The decision to exclude articles focused on a single health dimension or lifestyle interventions was made to ensure the study focused on service delivery models and practices supporting the physical health needs of adults with SMI comprehensively, making the findings more relevant for service redesign efforts.

### Search strategy

To locate scholarly articles, a medical librarian (TR) developed the core search strategy in MEDLINE in collaboration with the review team, then translated the search for use in other selected databases. Searches were conducted in the following four databases on July 19, 2023, and updated on June 6, 2024, using the same search strategy: MEDLINE (Ovid), Embase (Ovid), APA PsycInfo (Ovid), and CINAHL (EBSCO). The first section of the search strategy combined a robust “physical health care” concept comprised of database-specific subject headings, keywords in natural language, and advanced search operators with natural language strings of “integration” or “co-location” terms appearing within five words of terms related to mental healthcare or mental health conditions. The second section combined a “mental health care” concept with strings of “integration” or “co-location” terms appearing within five words of primary healthcare or physical health terms. The third section used subject headings that capture the programming or implementation aspects of integrated care, as well as “integration” or “co-location” keywords, which were then combined with subject headings from Sections One and Two. The results of all three sections were pooled and limited to publication years 2000 to present. No study type or language limits were applied. The full Medline strategy can be found in Supplementary Table S1.

### Evidence selection

Following the search, all identified citations were uploaded into Covidence where duplicate citations were removed. Titles and abstracts were screened by two independent reviewers, SZ and TM, for assessment against the inclusion criteria for the review. The full text of selected citations were assessed in detail against the inclusion criteria by two independent reviewers. At the beginning of these phases, the senior author, VS, reviewed an initial sample of 20 review decisions made by the two reviewers to ensure consistency. Reasons for exclusion of sources of evidence at full text review were recorded and reported in the scoping review. Disagreements between the reviewers at each stage of the selection process were resolved through discussion with the senior author and/or resolved by consensus.

### Data charting process and items

A data extraction template was developed by the research team to chart details about the included articles and relevant content. The domains of the data extraction form included: authors, publication year, country, article title, study type, target population/study setting, and key findings for all articles. Descriptions of the model and types of interventions were extracted. One research team member extracted the data from the included articles, which was reviewed by the senior author for accuracy and completeness. Given the nature of scoping reviews and the conceptual focus of this review, critical appraisal of article quality was not performed.

### Synthesis of results

Reverse integration initiatives, especially in North America, where most studies originated from, have typically followed the principles outlined by Wagner's Chronic Care Model for the treatment of adults with chronic illness and complex health needs. This model, focused on improving health outcomes through the provision of high quality, patient-centered and evidence-based care, has been central to integrated care delivery initiatives in the US, including collaborative care models in primary care settings. Service delivery models and practices were therefore examined using Wagner's Model as a guide to identify the essential elements that encourage high-quality physical health care and chronic disease management (66, 67). A descriptive process was used to identify and synthesize the most common elements within the service models and related outcomes.

## Results

**Figure 1:**
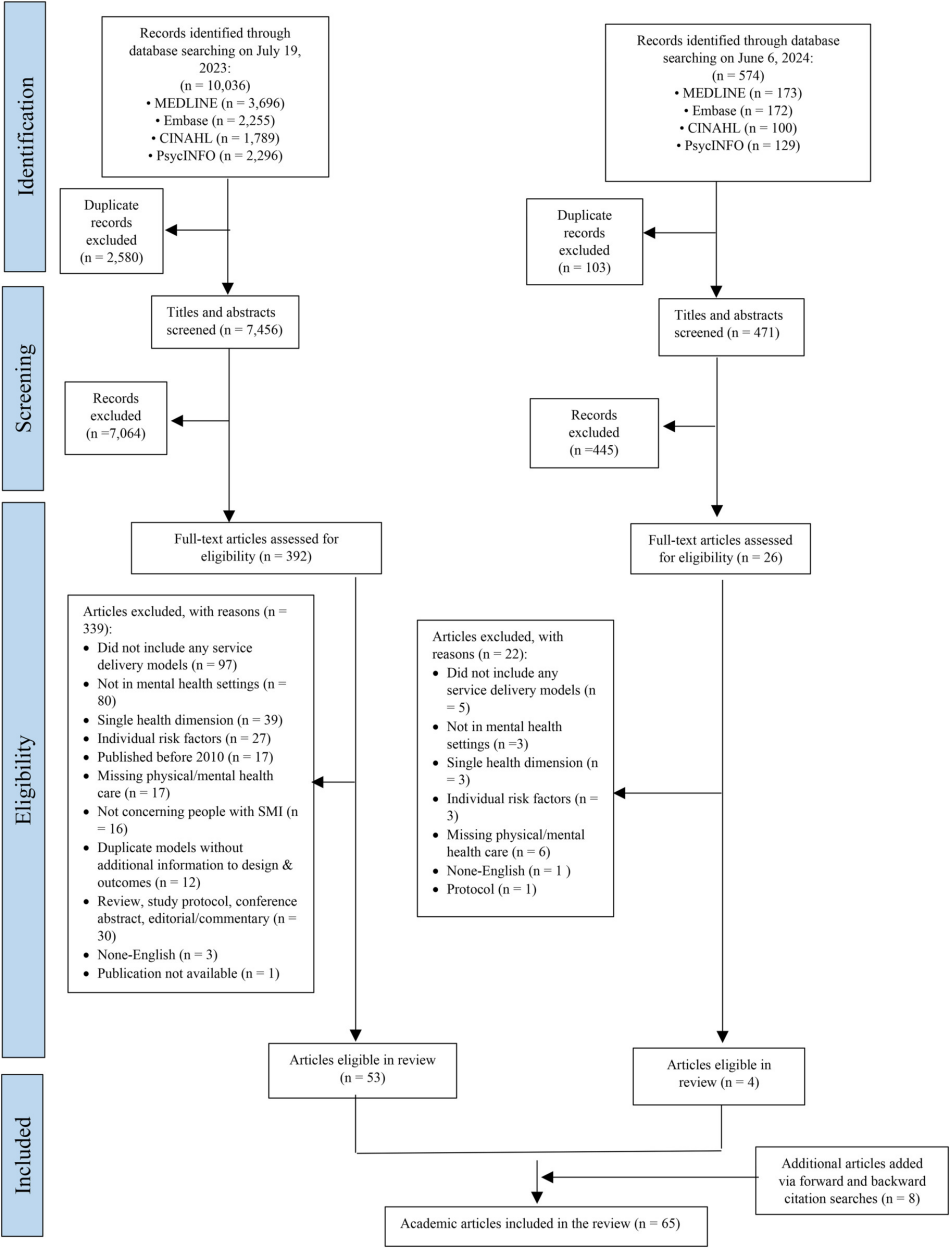
Study selection and exclusion process (PRISMA flow diagram).

A total of 10,610 records were identified through database searching across the two searches on July 19, 2023 and June 6, 2024 (10,036 records from the first search and 574 records from the second search). After removing duplicates, 7,927 titles and abstracts were screened. Following title and abstract screening, 418 articles were eligible for full-text review. Of these, 57 academic articles were included. Eight additional academic articles were included from forward and backward citation searches of the included articles, for a total of 65 academic articles (Table 1). The study selection process is presented in Figure 1. Most articles were from the United States (*n* = 54). Others were from Australia (*n* = 3), the United Kingdom (*n* = 3), Canada (*n* = 4), and Malta (*n* = 1).

**Table 1:** Overview of included studies.

Authors, year	Article title	Country	Study type
Rogers et al. 2016 (55)	A Randomized Clinical Trial Investigating the Effect of a Healthcare Access Model for Individuals with Severe Psychiatric Disabilities	USA	Randomized controlled trial (RCT)
Goh et al. 2016 (75)	A retrospective study of medical comorbidities in psychogeriatric patients	Australia	Retrospective descriptive study
Scharf et al. 2014 (89)	Evaluation of the SAMHSA Primary and Behavioral Health Care Integration (PBHCI) Grant Program: Final Report	USA	Multi-method program evaluation
Scharf et al. 2014 (86)	An Examination of New York State's Integrated Primary and Mental Health Care Services for Adults with Serious Mental Illness	USA	Qualitative study
Scharf et al. 2016 (95)	General Medical Outcomes From the Primary and Behavioral Health Care Integration Grant Program	USA	Quasi-experimental study
Breslau et al. 2018 (118)	Impact of a Mental Health Based Primary Care Program on Quality of Physical Health Care	USA	Quasi-experimental study
Breslau et al. 2018 (99)	Impact of a mental health based primary care program on emergency department visits and inpatient stays	USA	Quasi-experimental study
Krupski et al. 2016 (85)	Integrating Primary Care Into Community Mental Health Centers: Impact on Utilization and Costs of Health Care	USA	Quasi-experimental study
Druss et al. 2017 (49)	Randomized Trial of an Integrated Behavioral Health Home: The Health Outcomes Management and Evaluation (HOME) Study	USA	RCT
Johnson et al. 2022 (70)	Assessing the Long-Term Effectiveness of a Behavioral Health Home for Adults With Bipolar and Psychotic Disorders	USA	Longitudinal cohort study
Druss et al. 2020 (84)	Randomized Trial of a Mobile Personal Health Record for Behavioral Health Homes	USA	RCT
Pirraglia et al. 2012 (97)	Benefits of a primary care clinic co-located and integrated in a mental health setting for veterans with serious mental illness	USA	Longitudinal cohort study
Druss et al. 2010 (92)	A Randomized Trial of Medical Care Management for Community Mental Health Settings: The Primary Care Access, Referral, and Evaluation (PCARE) Study	USA	RCT
Druss et al. 2011 (48)	Budget impact and sustainability of medical care management for persons with serious mental illnesses	USA	RCT
Cabassa et al. 2015 (45)	What would it take? Stakeholders’ views and preferences for implementing a health care manager program in community mental health clinics under health care reform	USA	Qualitative study
Cabassa et al. 2019 (119)	“Treated me..Like I was family”: Qualitative Evaluation of a Culturally-Adapted Health Care Manager Intervention for Latinos with Serious Mental Illness and at Risk for Cardiovascular Disease	USA	Qualitative study
Ross et al. 2018 (74)	Can We Improve Physical Health Monitoring for Patients Taking Antipsychotics on a Mental Health Inpatient Unit?.	Canada	Quasi-experimental study
McGinty et al. 2018 (108)	An innovative model to coordinate healthcare and social services for people with serious mental illness: A mixed-methods case study of Maryland's Medicaid health home program	USA	Case study
Daumit et al. 2019 (47)	Care Coordination and Population Health Management Strategies and Challenges in a Behavioral Health Home Model	USA	Multi-method program evaluation
Murphy et al. 2020 (120)	Association Between the Maryland Medicaid Behavioral Health Home Program and Cancer Screening in People With Serious Mental Illness	USA	Quasi-experimental study
Annamalai et al. 2018 (88)	Establishing an Integrated Health Care Clinic in a Community Mental Health Center: Lessons Learned	USA	Descriptive study
Uga et al. 2017 (121)	Evaluation of a Model of Integrated Care for Patients With Chronic Medical and Psychiatric Illness	USA	Quasi-experimental study
Schmit et al. 2018 (56)	Examining the Effectiveness of Integrated Behavioral and Primary Health Care Treatment	USA	Quasi-experimental study
Pratt et al. 2013 (122)	Feasibility and Effectiveness of an Automated Telehealth Intervention to Improve Illness Self-Management in People With Serious Psychiatric and Medical Disorders	USA	Single-arm feasibility study
Gilmer et al. 2016 (96)	Implementation of Integrated Health Homes and Health Outcomes for Persons With Serious Mental Illness in Los Angeles County	USA	Longitudinal cohort study
Henwood et al. 2018 (73)	Integrated Primary Care in Assertive Community Treatment	USA	Descriptive study
Tse et al. 2022 (72)	Integrating Primary Care Into Assertive Community Treatment	USA	Quasi-experimental study
Carson Weinstein et al. 2011 (123)	Transforming assertive community treatment into an integrated care system: The role of nursing and primary care partnerships	USA	Descriptive study
Smali et al. 2022 (87)	A Continuum-Based Framework as a Practice Assessment Tool for Integration of General Health in Behavioral Health Care	USA	Descriptive study
Stevens and Sidlinger 2015 (71)	Integration of Primary Care into a Mental Health Center: Lessons Learned from Year One Implementation	USA	Descriptive study
Mangurian et al. 2022 (90)	Lessons Learned From a New Reverse-Integration Model to Improve Primary Care Screening in Community Mental Health Settings	USA	Descriptive study
Bartels et al. 2014 (68)	Long-term outcomes of a randomized trial of integrated skills training and preventive healthcare for older adults with serious mental illness	USA	RCT
Tepper et al. 2017 (91)	Mind the Gap: Developing an Integrated Behavioral Health Home to Address Health Disparities in Serious Mental Illness	USA	Quasi-experimental study
Storm et al. 2020 (124)	Peer Support in Coordination of Physical Health and Mental Health Services for People With Lived Experience of a Serious Mental Illness	USA	Qualitative study
Errichetti et al. 2020 (93)	Randomized Trial of Reverse Colocated Integrated Care on Persons with Severe, Persistent Mental Illness in Southern Texas	USA	RCT
Iturralde et al. 2022 (76)	Closing the Care Gap for People with Severe and Persistent Mental Illness: Collaborative Care, Telehealth, and Clinical Pharmacy	USA	Descriptive study
Iturralde et al. 2024 (77)	Telehealth Collaborative Care Led by Clinical Pharmacists for People With Psychosis or Bipolar Disorder: A Propensity Weighted Comparison With Usual Psychiatric Care	USA	Retrospective cohort study
Tajirian et al. 2023 (57)	Recommendations to Enhance Physical Health for Individuals with Severe Mental Illness in Canadian Healthcare Organizations	Canada	Descriptive study
Ungar et al. 2013 (109)	Reversed Shared Care in Mental Health: Bringing Primary Physical Health Care to Psychiatric Patients	Canada	Descriptive study
Lambert et al. 2017 (125)	Royal Australian and New Zealand College of Psychiatrists expert consensus statement for the treatment, management and monitoring of the physical health of people with an enduring psychotic illness	Australia	Delphi study
Mouko and Sullivan 2017 (126)	Systems for physical health care for mental health patients in the community: Different approaches to improve patient care and safety in an Early Intervention in Psychosis Service	UK	Longitudinal cohort study
Brown et al. 2020 (127)	The adaptation and implementation of the Health Improvement Profile to Australian standards in public mental health settings	Australia	Descriptive study
Xuereb et al. 2020 (128)	The implementation of a physical health checklist in a psychiatric forensic unit	Malta	Pre-post study
Malachowski et al. 2019 (107)	The Integrated Health Hub (IHH) Model: The Evolution of a Community Based Primary Care and Mental Health Centre	Canada	Qualitative study
Zatloff et al. 2021 (98)	Reverse Integration Pilot in a Public Safety-Net Hospital's Outpatient Behavioral Health Clinic	USA	Pre-post study
Chambers et al. 2023 (69)	Whole person care: Outcomes from a 5-year care model integrating primary care into a behavioral health clinic	USA	Pre-post study
Eldridge et al. 2011 (105)	A well-being support program for patients with severe mental illness: A service evaluation	UK	Descriptive study
Siantz et al. 2016 (129)	Implementation of Peer Providers in Integrated Mental Health and Primary Care Settings	USA	Qualitative study
Ma and Saw 2018 (102)	A Qualitative Study on Primary Care Integration into an Asian Immigrant-specific Behavioural Health Setting in the United States	USA	Qualitative Study
Wells et al. 2019 (130)	Integrating Primary Care Into Community Mental Health Centres in Texas, USA: Results of a Case Study Investigation	USA	Case study
Connor et al. 2018 (78)	Integrating physical health: What were the costs to behavioral health care clinics?	USA	Cost analysis
Ramanuj et al. 2018 (79)	Integrating Behavioral Health and Primary Care Services for People with Serious Mental Illness: A Qualitative Systems Analysis of Integration in New York	USA	Qualitative study
Scharf et al. 2013 (81)	Integrating primary care into community behavioral health settings: Programs and early implementation experiences	USA	Descriptive study
Breslau et al. 2021 (100)	Primary and Behavioral Health Care Integration Program: Impacts on Health Care Utilization, Cost, and Quality	USA	Collective case study
Bandara et al. 2020 (101)	The effects of the Maryland Medicaid Health Home Waiver on Emergency Department and inpatient utilization among individuals with serious mental illness	USA	Retrospective cohort study
McGinty et al. 2020 (131)	Effects of Maryland's Affordable Care Act Medicaid Health Home Waiver on Quality of Cardiovascular Care Among People with Serious Mental Illness	USA	Retrospective cohort study
Stone et al. 2020 (82)	The Policy Ecology of Behavioral Health Homes: Case Study of Maryland's Medicaid Health Home Program	USA	Case study
Tatreau et al. 2016 (132)	Cardiometabolic Assessment, Diagnosis, and Treatment of Chronic Medical Illnesses During an Inpatient Psychiatric Hospitalization: Colocated Medical Care Versus Treatment as Usual	USA	Cross-sectional retrospective study
Woltmann et al. 2024 (133)	Technologic and Nontechnologic Barriers to Implementing Behavioral Health Homes in Community Mental Health Settings During the COVID-19 Pandemic	USA	Qualitative study
Flanagan et al. 2024 (103)	Care integration goes Beyond Co-Location: Creating a Medical Home	USA	Qualitative descriptive study
Burner et al. 2024 (134)	Factors to Improve Reverse Integration: A Mixed Method Embedded Design Study	USA	Qualitative descriptive study
Utter et al. 2023 (135)	Integrating primary care services in outpatient mental health treatment facilities: National and state trends, 2015–2020	USA	Repeated cross-sectional study
Kogan et al. 2017 (106)	Challenges encountered in the conduct of Optimal Health: A patient-centered comparative effectiveness study of interventions for adults with serious mental illness	USA	Cluster-RCT
Nikolajski et al. 2022 (104)	Staff Perceptions of Barriers and Facilitators to Implementation of Behavioral Health Homes at Community Mental Health Provider Settings	USA	Qualitative study
Schuster et al. 2018 (94)	A Payer-Guided Approach To Widespread Diffusion Of Behavioral Health Homes In Real-World Settings	USA	Cluster-RCT

Articles included randomized controlled trials (*n* = 9), quasi-experimental studies (*n* = 10), longitudinal cohort studies (*n* = 4), qualitative studies (*n* = 12), descriptive studies, inclusive primarily of program descriptions (*n* = 12), pre-post study designs (*n* = 3), and case studies (*n* = 4). Please see Table 1 for description of all studies.

We present below a synthesis of our findings, including a description of the integrated care models identified, individual and system-level outcomes, implementation considerations, and associated costs.

### Service delivery models and clinical practices promoting physical health service delivery in mental health settings

The number of articles discussing integrated service delivery models and clinical practices increased over time: 21 articles were published from 2010 to 2016, and 44 were published from 2017 to June 6, 2024. Most of the service delivery models and clinical practices described were implemented in public sector community settings in the United States, such as community mental health centers and community-based behavioral health clinics serving adults with SMI. Commonly, initiatives integrated primary care physicians or nurse practitioners (NP) within outpatient behavioral health settings to establish on-site primary care clinics and support coordination with community-based primary health services (49, 56, 68–71). Efforts to integrate primary care physicians and NPs into Assertive Community Treatment (ACT) teams and inpatient psychiatric units to improve the assessment and treatment of physical health comorbidities in this population were also described (72–75). More recently, studies described a novel pharmacist-led collaborative care model leveraging telehealth and population-based care to support medication management, health screenings, and access to multidisciplinary services and community resources for adults with SMI in northern California (76, 77).

The services and practices discussed in the literature leverage different levels of the healthcare system, from macro-level policy levers and grant supports to deliver integrated care to micro-level direct practice changes. Macro-level initiatives, such as the Primary and Behavioral Health Care Integration (PBHCI) grant program in the US provided funding or financial incentives to integrate services for people living with SMI (78, 79). Meso-level initiatives focused on organizing and managing services at regional or organizational levels (80). These initiatives included optimizing leadership and strategic planning within organizations, regular monitoring and reporting of certain health indicators, and creating patient registries to track patients’ physical health needs (81, 82). These meso-level practices aimed to bridge the gap between broad policy directives and individual patient care and ensure that healthcare services are efficient and well-integrated. At the micro level, initiatives included designated healthcare professionals to support the physical health needs of patients within mental health settings and the development of personalized care plans.

The service delivery models and practices described have generally followed one or more of the elements outlined in Wagner's Chronic Care Model (CCM) for treating individuals with complex chronic health conditions (67). The core components of Wagner's CCM include patient self-management support, delivery system design, decision support, and clinical information systems, in addition to organizational commitment to safe, high quality care, and linkage to community resources. Models varied in their description of these components; seventeen of the 51 (33.3%) distinct models described in this review appeared to include four or more components of Wagner's CCM, whereas 20 (39.2%) appeared to include two or less. Among the six CCM components, the ones most commonly addressed include delivery-system redesign (100%), patient self-management support (52.9%) and use of clinical information systems (45.15%). Access to decision support was the component least likely to be discussed in these models (21.6%), although program descriptions were often limited and components of the model may have been missed.

Delivery-system redesign refers to redefining work roles for clinicians and staff to facilitate preventive care, as well as creating new positions as needed to support the care model (67, 83). The models emphasized screening and referral for the treatment of general medical conditions, designated primary care physicians, NPs, healthcare managers, or peer support specialists to monitor physical health issues regularly, and provided psychoeducation on illness prevention (68, 84–87). Patient self-management support, as part of comprehensive care models, involved empowering individuals to recognize and manage their symptoms (66, 67, 83). The support typically combined education and skills training, often facilitated by healthcare professionals such as nurses, NPs, and care managers. Some programs included care managers and peer providers, as well as wellness specialists offering health education on lifestyle factors such as weight loss, smoking cessation, diabetes management, and heart disease prevention (49, 85, 88).

Furthermore, to provide comprehensive physical and mental health care by a multidisciplinary team or across different teams, communication and information sharing among providers is crucial (66, 67). Some studies implemented and improved processes within electronic health records (EHRs) and reminder systems to ensure efficient documentation, information flow among clinicians, and timely reminders for care coordination. For instance, all PBHCI grantees were required to develop a registry/tracking system for physical health needs and outcomes (89). Other programs have enhanced EHR functionality with features like provider alerts for patient transitions, health status registries, standardized order sets and comprehensive discharge reports (74, 90, 91).

### Health indicator outcomes

Common physical health indicators assessed in the articles examined included blood pressure, blood glucose, cholesterol, and other cardiometabolic risk factors. Among the twelve studies that reported on physical health indicators, results were promising. Select findings are presented below, with study details described in Table 2.

**Table 2:** Description of integrated care delivery models/ clinical practices and associated outcomes.

Authors year	Description of the Integrated Care Model/Clinical Practice	Type of intervention	Study description	Key findings
Rogers et al. 2016 (55)	A nurse practitioner (NP) in a community mental health settings serving adults with SMI providing patient-centered care, lifestyle counseling, specialty care access, and coordination with primary care providers.	• Support of patient self-management • Delivery system redesign • Decision-making support	This randomized controlled trial included 200 participants, who were randomly assigned to the intervention group (*n* = 94) or usual care (*n* = 106) and followed for 12 months.	Individuals receiving NP services experienced significant improvements in Continuity of Care (F = 2.73, df = 3,430, *p* = .04) and the Community Orientation of the primary care provider (F = 2.71,df = 3,412, *p* = .05). There were no differences in exercise, nutrition and wellness outcomes.
Goh et al. 2016 (75)	A medical resident in an inpatient psychogeriatric unit managing medical comorbidities in psychiatric patients 65 and older.	Delivery system redesign	Retrospective file (*n* = 165) audit analyzed admissions to assess medical comorbidities and interventions.	91.5% of inpatients had at least one medical comorbidity. Medical assessments increased from 24% to 53% with the introduction of medical resident [*χ*^2^ (2) = 15.17, *P* = 0.001]. The increase did not affect rates of emergency medical transfers, geriatric evaluation visits, or changes in non-psychiatric drug treatments.
Scharf et al. 2014 (89)	Physical and Behavioral Health Care Integration (PBCHI) grantees received $500,000 annually to coordinate access to primary care, including four core features: screening/referral for physical health needs, developing a registry/tracking system, care management, and prevention/wellness support.	• Support of patient self-management • Decision-making support • Use of clinical information systems • Delivery system redesign	This article describes an evaluation of the PBHCI grants program, including document review, program data and comparative study of 3 PBHCI clinics and 3 control clinics.	PBHCI programs varied in structure and integration features. PBHCI consumers, compared to controls, showed improvements in some (e.g., diastolic blood pressure, total and LDL cholesterol, blood glucose), but not all physical health indicators examined (e.g., systolic blood pressure, body mass index, hemoglobin A1c, triglycerides, smoking). Access to primary care and integrated care was not clearly associated with physical health outcomes.
Scharf et al. 2014 (86)	• Three initiatives aimed at integrating care for adults with SMI in New York State (NYS). • PBHCI programs: see Scharf, Eberhart, et al. 2014 (86) • Medicaid Incentive Program offered financial incentives for mental health clinics to expand their billable services offering health monitoring or health monitoring and health physicals. • Medicaid Health Homes included integrated networks of diverse healthcare providers managed by lead organization, focusing on coordinated, multidisciplinary care for patients with complex medical needs.	PBHCI programs: see Scharf et al. 2014 (86) Medicaid Incentive Program: a market incentive mechanism to promote integrated care. Medicaid Health Homes: • Delivery system redesign • Linkage to community resources	Descriptive study of three initiatives, leveraging data from site visits to nine mental health clinics and surveys with 22 mental health clinic administrators and 34 associated professionals.	PBHCI clinics were more likely to develop an integrated care culture, use registries and offer on-site comprehensive services. Medicaid Incentive clinics had limited scope, while Mental Health Homes relied on case managers and networks of organizations to offer access to primary care, focusing on care coordination. Effective clinics leveraged connections with community programs, data systems, information sharing, and strong leadership. Challenges included licensing requirements, infrastructure, information sharing, and sustainability.
Scharf et al. 2016 (95)	PBHCI program: see Scharf et al. 2014 (89)	See Scharf et al. 2014 (89).	This quasi-experimental study used a difference-in-differences design to compare changes in general medical health between consumers in PBHCI clinics (*n* = 322) and control clinics (*n* = 469) over approximately a year.	PBHCI consumers showed statistically significant improvements in total cholesterol, LDL cholesterol, and HDL cholesterol compared to control consumers. The adjusted mean reduction in total cholesterol was 36 mg/dl (*p* < 0.01), in LDL cholesterol 35 mg/dl (*p* < 0.001), and the increase in HDL cholesterol 3 mg/dl (*p* < 0.05)
Breslau et al. 2018 (99)	PBHCI program: see Scharf et al. 2014 (89). Seven New York City (NYC) outpatient mental health clinics with PBHCI programs (4 programs implemented in wave 1, and 3 implemented in wave 2).	See Scharf et al. 2014 (89).	Medicaid claims data of PBHCI patients from 2 waves of implementation (*n* = 8,603) and control participants (*n* = 24,581) from 40 New York City (NYC) clinics without primary care services were used to assess impact of PBHCI on quality of physical health care.	For wave 1 participants, there was a statistically significant (*p* < 0.0001) increase in the odds of receiving metabolic monitoring among antipsychotic users in PBHCI clinics relative to controls, and no differences on the odds of having an outpatient medical visit or diabetes monitoring. For wave 2, there were no significant differences between the PBHCI and control groups for any of the quality measures examined.
Breslau et al. 2018 (99)	PBHCI programs: see Scharf et al. 2014 (89) Seven New York City (NYC) outpatient mental health clinics with PBHCI programs (4 programs implemented in wave 1, and 3 implemented in wave 2).	PBHCI programs: see Scharf et al. 2014 (89)	Medicaid claims data of PBHCI patients from 2 waves of implementation (*n* = 8,603) and control participants (*n* = 24,581) from 40 New York City (NYC) clinics without primary care services were used to assess impact of PBHCI on emergency department (ED) visits and hospitalizations.	Hospitalizations for medical conditions increased in PBCHI clinics compared to control in both waves (OR = 1.21 for Wave 1, OR = 1.33 for Wave 2). ED visits for behavioral health conditions decreased in PBCHI clinics relative to controls in Wave 1 (OR = 0.89), but not in Wave 2. There were no other significant differences in healthcare utilization between PBCHI and control clinics.
Krupski et al. 2016 (85)	PBHCI program: see Scharf, et al. 2014 (89). An advanced NP and nurses coordinating primary and mental health care in 2 community mental health centers serving vulnerable and homeless populations in King County, Washington. Medical staff handled referrals, and peer counselors led wellness programs under nurse supervision.	• Support of patient self-management • Use of clinical information systems • Delivery system redesign • Support from the health care organization • Linkage to community resources	The study compared outcomes of adults enrolled in the PBHCI centers (clinic 1, *n* = 373; clinic 2, *n* = 389) to propensity matched controls from the same sites. Clinic 1 had a 10 year history of providing integrated care, while Clinic 2 began integrating care with the PBHCI grant.	Outcomes (Clinic 1): Increased outpatient care (*p* < .003), decreased inpatient admissions (*p* = .04), trend for lower inpatient costs ($217.68, *p* = .06). Outcomes (Clinic 2): Increased outpatient care (*p* < .001), no significant inpatient cost changes.
Druss et al. 2017 (49)	The Behavioral Health Home (BHH) had a NP and a nurse care manager, supervised by the health center's director. They targeted cardiometabolic risks (blood pressure, glucose level, cholesterol level) and integrated health records with mental health teams serving adults with SMI. Patients received health education and logistical support to attend their medical appointments	• Support of patient self-management • Use of clinical information systems • Delivery system redesign	This single-blinded randomized controlled trial involved 447 adults with SMI and one or more cardiometabolic risks randomized to the BHH group (*n* = 224) or usual care (*n* = 233), which included providing participants a summary of their lab results and encouraging to see their medical provider.	BHH patients had significant improvements in cardiometabolic care (from 67% to 81%), diabetes, and hypertension care (both *p* < 0.001), with higher likelihoods of receiving appropriate medications for diabetes and hypertension. BHH patients also showed greater improvements in preventive services (from 36% to 56%) and care alignment with the chronic care model (from 2.2 to 3.6), both significantly better than usual care (*p* < 0.001).
Johnson et al. 2022 (70)	The BHH, serving patients with psychotic and bipolar disorder in an urban setting, provided referrals and co-located services with primary care, involving a NP and care manager, supported by regular meetings.	• Support of patient self-management • Use of clinical information systems • Delivery system redesign • Linkage to community resources	EHR data was used to compare BHH participants (*n* = 413) with non-BHH participants (*n* = 1,929) in regards to health care utilization and chronic disease management 3.5 years post-BHH implementation	BHH participants, compared to controls, had a significant increase in primary care visits per month (+0.18 visits/month, *p* < 0.01), a significant decrease in emergency department (ED) visits per month (−0.031 visits/month, *p* < 0.01) and more general medical health outpatient visits per month (+0.055, *p* < 0.01). BHH participation was associated with significant reduction in hemoglobin A1c levels (−0.29%, *p* < 0.05), but no differences in LDL cholesterol values.
Druss et al. 2020 (84)	The Mobile Personal Health Record (mPHR) app tracked health data and goals among BHH participants with SMI and one or more cardiometabolic risk factors, with certified peer specialists trained as clinical technology specialists assisting participants in using the app.	• Support of patient self-management • Use of clinical information systems • Delivery system redesign	This randomized controlled trial compared quality of medical care among participants randomized to receive the mPHR app (*n* = 156) or usual care (*n* = 155) over 12 months.	Participants in the intervention group received 70% of indicated cardiometabolic and preventive services at both baseline and 12-month follow-up, while the usual-care group showed a slight but statistically significant decline from 71% to 67% (F = 4.18; df = 1, 309; *p* = 0.04).
Pirraglia et al. 2012 (97)	The Serious Mental Illness Primary Care Clinic (SMIPCC) colocated and integrated in a mental health outpatient program targeting veterans with SMI with poor primary care engagement and at least one chronic medical condition, operated one session per week staffed by a primary care provider and a patient care assistant.	Delivery system redesign	This longitudinal cohort study involved chart reviews of veterans (*n* = 97) with SMI 6 months prior to enrollment and 6 months in the year following enrollment to a co-located primary care clinic.	Enrollment in the SMIPCC program was associated with a significant increase in primary care visits (median increased from 0 to 2, *p* < 0.001) and higher goal attainment for several health metrics: Blood pressure: AOR = 2.16 (95% CI, 1.47–3.18) LDL cholesterol: AOR = 1.60 (95% CI, 1.10–2.34) Triglycerides: AOR = 1.64 (95% CI, 1.06–2.51) BMI: AOR = 1.81 (95% CI, 1.29–2.54) Changes with regard to goal attainment for high-density lipoprotein cholesterol and HbA1c were not significant.
Druss et al. 2010 (92)	The Primary Care Access, Referral, and Evaluation (PCARE) model used care managers, coordinated by two full-time nurses, to support self-management, advocate for patients, maintain provider lists for population-based medical care management, and enroll uninsured patients. The target population was economically disadvantaged adults with SMI in an urban community mental health center.	• Support of patient self-management • Delivery system redesign • Linkage to community resources	A randomized controlled trial comparing the quality of care among PCARE (*n* = 205) and usual care participants (*n* = 202) at 6 and 12 months.	At 12-months, the PCARE group received significantly more recommended preventive services (58.7% vs. 21.8%, *p* < 0.001) and had better outcomes in cardiometabolic care and primary care provider access. They also showed significant improvement in the SF-12 Mental Component Score (8.0% improvement vs. 1.1% decline, *p* = 0.008), and had lower Framingham Cardiovascular Risk Scores compared to the control group (6.9% vs. 9.8%, *p* = 0.02).
Druss et al. 2011 (48)	PCARE model. See Druss et al. 2010 (92).	See Druss et al. 2010 (92).	Study evaluated two-year outcomes, costs, and financial sustainability of a medical care management intervention in community mental health settings via chart reviews of PCARE participants and interviews.	At 2 years, the intervention group, compared to usual care, showed sustained improvements in quality of primary care preventive services, cardiometabolic care, and mental health-related quality of life (all at *p* < 0.001), with a reduced total cost by $932 by year 2. The program was not sustainable without grant funding as only 40.5% of participants had health insurance.
Cabassa et al. 2015 (45)	An adaptation of the PCARE model using social workers instead of RNs to deliver the intervention in outpatient mental health clinics in Northern Manhattan serving predominantly Hispanic patients with SMI. See Druss et al. 2010 (92) for the PCARE model.	See Druss et al. 2010 (92).	20 stakeholders (mental health providers, primary care providers, administrators and consumer advocates) participated in semi-structured qualitative interviews to assess the feasibility and acceptability of implementing an adapted PCARE model at a public mental health outpatient clinic.	Stakeholders valued PCARE's care coordination, physical health focus, and liaison role of the health care manager. Concerns included integrating into routine care and staff workloads. A blend of implementation strategies was recommended (e.g., financial, restructuring, cultural adaptation) to move this intervention into practice.
Cabassa et al. 2019 (119)	Bridges to Better Health and Wellness (B2BHW) healthcare managers connected patients of a public outpatient mental health clinic in New York City to primary care, ensured patients’ medical information was shared across levels of care, monitored patients’ health, and alerted providers when preventive primary care services were needed.	• Support of patient self-management • Delivery system redesign • Decision-making support	The study included a survey (*n* = 29) and 3 focus groups (*n* = 16) of participants receiving the B2BHW intervention.	Participants valued the respectful, supportive relationships with healthcare managers, the health education they received, as well as care coordination and patient activation which reflected cultural norms and addressed key barriers to care.
Ross et al. 2018 (74)	Implementation of a standardized electronic order set in an acute inpatient psychiatry ward promoting specific health investigations for patients prescribed a regularly scheduled antipsychotic medication for 3 or more days.	• Use of clinical information systems • Delivery system redesign	A chart audit focused on patients prescribed antipsychotics for 3 or more days before (*n* = 96) and after implementation (*n* = 190) of a standardized order set.	The implementation of the standardized electronic order set significantly improved (*p* < 0.05) physical health monitoring rates for blood glucose, lipids, ECG, and thyroid-stimulating hormone (TSH) in patients with SMI on antipsychotic medications (monitoring rates for blood glucose, lipids ECG and TSH improved from 31%, 36%, 51% and 71% to 96%, 64%, 87% and 86% respectively). Intervention rates for abnormal physical health results remained low.
McGinty et al. 2018 (108)	Maryland's Medicaid Behavioral Health Home (BHH) Program included a director, nurse care managers, and primary care consultants to provide individualized care plans, care coordination, health promotion, transitional care, support services, and community referrals to eligible participants followed by psychiatric rehabilitation programs ((PRPs) serving adults with SMI in Maryland.	• Support of patient self-management • Use of clinical information systems • Delivery system redesign • Linkage to community resources	This case study, using interviews with 72 BHH implementation leaders and a survey of 627 frontline staff, examined the implementation of Maryland's BHH program in 46 out of 48 active programs during the study period.	BHH program structure varied across sites. 93% of staff supported integrating somatic health services in PRPs, though 37% preferred focusing more on social services, indicating tension in service priorities. Despite good organization fit, implementation challenges included health IT usability, population health management capacity and coordination with external providers.
Daumit et al. 2019 (47)	Maryland Medicaid Behavioral Health Home (BHH) Program, see McGinty et al. 2018 (108).	See McGinty et al. 2018 (108).	The study, using interviews with 72 BHH leaders and a survey of 627 frontline staff from 46 of 48 active programs, examined providers’ perceptions of and capacity to address BHH implementation barriers in community health centers in Maryland.	Population health management was challenged by tensions with direct clinical care provision, limited staff experience and state regulations for service delivery. While engaging primary care providers (PCPs) was the main barrier to care coordination, health information technology usability and staffing were barriers to both care coordination and population health management.
Murphy et al. 2020 (120)	Maryland Medicaid Behavioral Health Home (BHH) Program, see McGinty et al. 2018 (108).	See McGinty et al. 2018 (108).	Using administrative data, the study examined the association between cancer screening and enrollment in a BHH (*n* = 3,298, 27%) vs. no enrollment (*n* = 8,878, 73%). Participants were adults in Maryland's psychiatric rehabilitation programs for who were eligible for cervical (*n* = 6,811), breast (*n* = 1,658), or colorectal cancer screening (*n* = 3,430).	BHH enrollment was associated with increased cervical (odds ratio [OR] = 1.20, 95% confidence interval [CI] = 1.07–1.35; *p* = 0.002)) and breast (OR = 1.30, 95% CI = 1.06–1.59; *p* = 0.01) cancer screening rates but not colorectal cancer screening. Predicted annual screening rates for BHH-enrolled individuals were higher but remained suboptimal, at 31% for cervical and 28% for breast cancer.
Annamalai et al. 2018 (88)	The Connecticut Mental Health Center (CMHC) Wellness Center is an on-site primary care clinic formed in partnership between the CMHC and a FQHC. The Wellness Centre provides health promotion programs for prevention and management of chronic health conditions. For specialty services, patients are referred to community or hospital-based practices.	• Support of patient self-management • Delivery system redesign	The authors describe the development of the on-site clinic and lessons learned during implementation.	The authors highlighted the importance of funding and leadership support, of developing a shared work culture and commitment between participating organizations, ongoing data monitoring, and hiring staff comfortable with integrated care and SMI challenges.
Uga et al. 2017 (121)	An integrated care clinic providing primary care and psychiatric services by dually-trained internists/psychiatrists, enhancing care coordination and communication for patients with chronic comorbid physical and psychiatric illnesses in academic outpatient clinics.	• Delivery system redesign • Use of clinical information systems	The study compared the quality of life, care satisfaction, and care utilization in participants from an integrated medicine and psychiatry clinic (*n* = 64) and participants from separate internal medicine and psychiatry clinics (*n* = 52) within the same institution.	Patients treated in the integrated clinic reported greater satisfaction with care for both medical (*p* < 0.01) and psychiatric (*p* < 0.01) illnesses, though quality of life was similar between the groups. There was a non-significant trend toward fewer emergency room visits and fewer hospital stays for the integrated care group.
Schmit et al. 2018 (56)	An integrated behavioral and primary healthcare (IBPH) approach including monthly or bimonthly medical services by primary care physicians or NPs within a rural community mental health agency serving adults with SMI, facilitated by specialized case managers who ensured care continuity and provided preventive health services.	• Support of patient self-management • Delivery system redesign	A quasi-experimental pre–post study using health record data to compare the effectiveness of IBPH (*n* = 98) vs. behavioral treatment as usual (TAU) (*n* = 98) in improving holistic functioning over 12 months.	Participants in the IBPH group experienced a 24 times greater improvement in overall functioning compared to TAU, based on profile analysis of 5 mean difference scores capturing different domains of holistic functioning.
Pratt et al. 2013 (122)	A tailored automated telehealth intervention supported by nurse offering healthcare management monitoring responses, providing feedback, and facilitating follow-up care for adults with SMI and chronic medical conditions served by a community mental health center.	• Support of patient self-management • Use of clinical information systems • Delivery system redesign	This single arm pilot trial (*n* = 70) examined the feasibility, acceptability and potential effectiveness of the intervention over 6 months.	The intervention was deemed acceptable and feasible, with 89% of those engaged participating in >70% of sessions. Participation was associated with improvements in self-efficacy in managing depression [*t*(59) = 2.33; *p* = 0.023] and blood pressure [*t*(59) = 2.81; *p* *=* 0.008] and better understanding of their medical condition.
Gilmer et al. 2016 (96)	The study examined 2 models of integrated health homes in Los Angeles County: Model 1: Five mobile Housing First (HF) teams using ACT, with a FQHC providing general medical services and integrated field-capable clinical care. Model 2: Five integrated clinics pairing community mental health centers with FQHCs. Both models aimed to offer coordinated primary, mental health, and substance use care within an integrated multidisciplinary team for adults with SMI.	• Support of patient self-management • Delivery system redesign • Support from the health care organization • Use of clinical information systems • Linkage to community resources	This study combined site visits to assess the degree of integration in each setting with longitudinal program data on physical health, mental health recovery, and chronic condition screenings.	Participants in better integrated programs, compared to those in less integrated programs, showed greater improvements in physical health status and mental health recovery, higher screening rates for common health conditions and greater reductions in hypertension, but an increase in prediabetes and diabetes (all at *p* < 0.01). Highly integrated programs had better scores for use of peer support, engaging participant social supports, continuous quality improvement, care coordination, and care management.
Henwood et al. 2018 (73)	Five Housing First (HF) teams using ACT in Los Angeles County paired with FQHCs for general medical care using one of tree approaches: collocating ACT at a FQHC, integrating a primary care provider in ACT, or having primary care providers divide their time between ACT and FQHC.	See Gilmer et al. 2016 (96).	Site visits were conducted to examine how partnerships with FQHCs enable the provision of integrated care within HF ACT teams and assess screening rates for blood pressure, cholesterol and blood glucose.	Screening rates varied across the 5 programs. The type of partnership or model may be less important than effective communication between staff in determining integration success and better screening rates.
Tse et al. 2022 (72)	Primary care NPs in a postgraduate fellowship program in a FQHC assigned to five ACT teams in New York City for 2 days per month conducting field visits, joining wellness groups, managing walk-in visits, and consulting with ACT psychiatrists.	Delivery system redesign	Focus groups explored the care experiences of 20 staff members and 16 ACT participants from 5 ACT teams. Screening rates for hemoglobin A1c and cholesterol among the 5 ACT team participants (*n* = 305) over time were compared with control participants served by an ACT team with no integrated primary care (*n* = 73).	There was improved primary care engagement and an increase in cholesterol (from 16% to 36%) and hemoglobin A1C screenings (from 12% to 34%) for ACT participants receiving integrated care (*p* < 0.001), although field visits were found to be an inefficient use of time for NPs.
Carson Weinstein et al. 2011 (123)	Two Housing First ACT teams in Philadelphia with embedded primary care physician one day/week, nurse training in Guided Care, and enhanced electronic documentation system delivering integrated care on-site, in homes, and at the physician's hospital-based medical home.	• Use of clinical information systems • Delivery system redesign • Decision-making support • Linkage to community resources	A program description	By embedding primary care providers, redefining the ACT nurse's role to include a broader range of health care responsibilities and providing nurses with training and guidelines, ACT teams can effectively deliver integrated health care.
Smali et al. 2022 (87)	Use of a General Health Integration (GHI) framework to assess practices in community behavioral health settings in New York and identify opportunities to advance integration of general health care.	• Support of patient self-management • Delivery system redesign • Decision-making support • Linkage to community resources	Eleven behavioral health clinics in New York City serving 7,143 clients were introduced to the framework through webinars. Participants completed an online survey to assess their integration status and provide feedback on the tool's utility in guiding integrated care.	Clinics identified strengths in trauma-informed care, social service linkages, self-management support and quality improvement. Improvement opportunities were identified in screening and referral, evidence-based treatments, care teams and sustainability. Clinics reported positive experiences using the framework.
Stevens and Sidlinger 2015 (71)	A primary care clinic in a behavioral community mental health center serving adults with SMI, offering illness screening, exams and disease management.	• Support of patient self-management • Delivery system redesign • Support from the health care organization	Description of program implementation through a partnership between a local hospital, a school of nursing and a behavioral community health center.	In the first year, 325 patients were served with over 800 visits, and a reported productivity in face to face patient encounters of 99.85%.
Mangurian et al. 2022 (90)	The intervention (CRANIUM) added a remote primary care consultant and a peer navigator to an urban mental health team, used a registry to track lab results from EHRs, and focused on screening and evidence-based treatment.	• Use of clinical information systems • Delivery system redesign • Decision-making support	Electronic health records data, provider feedback, and patient screening rates, assessed feasibility of implementation, while cost analysis and process mapping assessed resource use.	High provider adoption and satisfaction with 7% increase in metabolic screening rates and increased HIV testing (from 1% to 17%). Limited sustainability due to short-term funding and difficulty maintaining patient registry.
Bartels et al. 2014 (68)	The HOPES (Helping Older People Experience Success) program combined 12 months of weekly psychosocial skills training with biweekly community practice trips and monthly visits from an embedded nurse for screenings, advance care planning, and primary healthcare coordination, followed by 1-year maintenance phase of monthly sessions.	• Support of patient self-management • Delivery system redesign • Decision-making support • Linkage to community resources	A randomized controlled trial engaging two community mental health centers to compare HOPES (*n* = 90) to usual care (*n* = 93) for adults with SMI >50years at 1, 2, and 3 years post randomization. Assessments of functioning, symptoms and service use involved self-reports, case manager ratings, and performance-based tasks.	HOPES was associated with improved community living skills [F(2,151) = 5.10, *p* = 0.007], lower psychiatric symptom severity, and higher rates of preventive healthcare screenings (eye exams, hearing tests, mammograms and Pap smears) at 3 years, with the greatest between-group difference found for receipt of mammograms and Pap smears (NNT = 5.5 and 3.5, respectively). Further, there was an almost twofold difference between HOPES and control participants for completing advance directives (NNT = 3.6). There were no differences in the number of medical conditions, health status or acute care use at 3 years.
Tepper et al. 2017 (91)	An integrated behavioral health home (BHH) focused on adults with psychotic or bipolar disorder, expanding on-site medical care and health promotion, improving electronic health record (her) functionality, adding NP, care manager, and program manager, and offering team-based care with a focus group therapy, social inclusion, on disease screening and monitoring and population management.	• Support of patient self-management • Delivery system redesign • Use of clinical information systems • Linkage to community resources	This quasi-experimental study used electronic health record date to compare outcomes among 424 BHH patients and a propensity matched control group of 1,521 individuals from the same health system a year before and after the BHH intervention.	ED visits significantly decreased among BHH patients (from 1.45 to 1.19 visits) compared to the control group, whose ED visits increased (from 0.99 to 1.16, *p* = 0.014). Psychiatric hospitalizations per capita significantly decreased for BHH patients (from 0.22 to 0.10) but remained stable in the control group (*p* = 0.002). There were no significant differences in medical hospitalizations. HbA1c screening rates increased significantly more among BHH patients (from 49% to 64%), compared to the control group (from 40% to 46%, *p* = 0.026) but there were no differences in lipid monitoring and no differences in changes in metabolic monitoring parameters among patients with diabetes.
Storm et al. 2020 (124)	Peer providers in six community mental health centers, using their personal experiences to connect individuals with essential resources and services, and coordinate physical and mental health care.	• Support of patient self-management • Delivery system redesign • Linkage to community resources	Qualitative study engaging 23 mental health professionals and 5 peer specialists in semi-structured interviews exploring peer specialists roles in coordinating physical and mental health care.	Peer specialists assisted adults with SMI through advocacy, practical and emotional support, connection to resources, health care visit preparation, mutuality and sharing of experiences. Securing funding and sustaining the same group of peer specialists over time was challenging.
Errichetti et al. 2020 (93)	A multidisciplinary team including primary care physician, nurses, dietician, medical support staff, and care coordinators, collocated in behavioral health clinic in Southern Texas serving adults with SMI who lacked primary care provider.	• Delivery system redesign • Use of clinical information systems	Randomized trial evaluating the effect of integrated care (at least two visits with a primary care provider and one visit with a chronic care nurse or dietician) on adults with SMI and co-morbid chronic illness. Health outcomes, including blood pressure, HbA1c, BMI, cholesterol, and depressive symptoms, were measured at baseline, 6 and 12 months in participants in the intervention (*n* = 249) and usual care groups (*n* = 167).	Intervention participants showed significantly lower systolic blood pressure (adjusted mean difference −3.86, *p* = 0.04) and average HbA1c (adjusted mean difference −0.36, *p* = 0.001) at 12 months compared to controls. There were no significant differences in diastolic blood pressure, body mass index, cholesterol or depressive symptoms.
Iturralde et al. 2022 (76)	A population-based treatment model utilizing advanced practice clinical pharmacists as care continuity navigators for patients with serious mental illness (SMI). The program, including pharmacist-led collaborative care, population management and telehealth within a large health care system in Northern California, provided individuals with SMI medication management, health screenings, and access to multidisciplinary services and community resources.	• Support of patient self-management • Delivery system redesign • Use of clinical information systems • Linkage to community resources • Support from the health care organization	The authors describe the development of a novel Population Care Model for adults with SMI.	87% of outreached patients had an intake assessment, and of those, 73% attended a follow-up appointment. Program received high patient satisfaction ratings (93/100).
Iturralde et al. 2024 (77)	See, Iturralde et al. 2022 (76)	See, Iturralde et al. 2022 (76)	Using electronic health record data, the study compared program enrollees (*n* = 968) with SMI at 6 demonstration sites (Population Care- PC) to propensity matched patients with SMI at 6 non-program sites (Usual Care- UC) (*n* = 8,339). Difference-in-difference analyses assessed changes in outcomes from 12 months pre- to 12 months post-enrollment. Primary outcomes included optimal psychotropic medication adherence, guideline-recommended glycemic screening, annual psychiatrist visits, and emergency department use.	PC participants showed significantly greater achievement of psychotropic medication adherence (ARD = 6.4; 95% CI = 2.5–10.4) and glycemic screening (ARD = 9.3; 95%CI = 5.0–13.7) from pre- to post-enrollment compared to UC. Annual psychiatrist visits decreased more among PC compared to UC participants (ARD = −5.8; 95% CI = −10.0 to −1.5). PC participants showed an increase in the receipt of lipid tests (ARD = 13.0; 95% CI = 8.1–17.9) and increased EKG evaluations (ARD = 6.8; 95% CI = 2.0–11.5). Pre to post enrollment changes in mental health related ED use, hospitalization, and primary care visits were not significantly different between PC and UC.
Tajirian et al. 2023 (57)	Strategies to advance integrated care in a large academic psychiatric hospital in Toronto, Canada, included a mobile nursing team, daily hospitalist presence, educating healthcare professionals, developing hospital partnerships to minimize external transfers, use of protocols and order sets and optimizing the EHR for better outcome measurement and communication.	• Use of clinical information systems • Delivery system redesign • Decision-making support • Support from the health care organization	Description of development and implementation of integrated care strategy.	Health professional education and upskilling, strategic partnerships with general hospitals, enhancing HER functionality and leadership support deemed essential for advancing integrated care in mental health setting.
Ungar et al. 2013 (109)	A primary care physician and an ACT nurse are available for appointments at a health clinic co-located with the Mental Health Community Day Treatment, Outpatient, and Outreach Services of an academic hospital in Toronto, Canada, one morning per week.	• Delivery system redesign • Support from the health care organization	Program description The pilot involved 51 patients: 25 were seen during the first three months (January–March 2010), and 26 additional patients over the next seven months (April–October 2010). Over the three months, 50 office visits were conducted, comprising 25 first visits and 25 repeat visits, with six no-shows.	The program successfully engaged 51 patients with SMI in 51 first and 109 repeat visits offering assessment, treatment and referrals to appropriate health services. Key challenges included securing financial and administrative support, addressing perceptions of increased costs, and overcoming institutional resistance to integrating services across departments.
Lambert et al. 2017 (125)	Royal Australian and New Zealand College of Psychiatrists expert consensus statement for the management of the physical health of people with a psychotic illness offering assessment and follow up checklists for providers, and clarifying practice expectations for providers and organizational leaders.	• Support of patient self-management • Delivery system redesign • Decision-making support	Delphi method used to reach consensus on strategies for physical health management of adults with SMI, engaging 55 clinicians, 21 carers, and 20 consumers.	Endorsed strategies included need for partnerships, training and upskilling, support with screening and self- management and development of key performance indicators.
Mouko and Sullivan 2017 (126)	Strategies to increase physical health assessments in an Early Intervention in Psychosis program in Bath and north East Somerset included adding education and data collection tools, a mobile physical health clinic, and reminder letters to complete health checks.	• Use of clinical information systems • Delivery system redesign • Decision-making support	Phased improvement intervention evaluating the effect of various strategies in improving rates of physical health monitoring. Four intervention phases were tested: increased awareness and clinical tools, mobile health clinics, GP reminder letters, and a combination of these approaches.	After phases 1–3, physical health checks improved from 0% to 43.9%, blood tests from 6.3% to 74.4%, and ECGs from 3.8% to 45.1%. Phase 4 showed sustained success, with 48% of patients completing all health checks, blood tests (64.6%), and ECGs (92.3%). Mobile health clinics had physical health check completion rates of 60%, and blood tests in 70%.
Brown et al. 2020 (127)	The adapted Health Improvement Profile (HIP) for Australia, assessing the physical health of mental health service users, including vaccination, smoking cessation, diet, exercise, sleep, substance use, and routine health exams, implemented in a 50 bed psychiatric inpatient unit and a community mental health center in Australia.	• Delivery system redesign • Decision-making support	Evaluation of the implementation of the adapted HIP, including HIP completion rates and clinician (*n* = 29) and service user (*n* = 12) surveys of their experiences using the adapted HIP.	HIP forms were completed for 54% (*n* = 137) of inpatient users and 15% (*n* = 34) of community service users. Clinicians and service users found the HIP to be an acceptable screening tool.
Xuereb et al. 2020 (128)	Introduction of a physical health checklist for Forensic Unit admissions in a hospital in Malta, including vital signs, cardiovascular, respiratory, abdominal, and neurological exams.	Delivery system redesign	Chart audit of comprehensiveness of physical health assessments before (*n* = 48) and after (*n* = 41) checklist implementation.	Checklist implementation resulted in an increase in physical health documentation from 65% to 98% of consecutive admissions. Documentation of cardiovascular, respiratory, and gastrointestinal exams improved from 27% to 98% and neurological exams from 17% to 90%.
Malachowski et al. 2019 (107)	The Integrated Health Hub (IHH) in a community mental health center in Ontario, Canada, featuring a Nurse Practitioner (NP) led primary care clinic and as needed psychiatric consultations providing comprehensive care to adults with mental illness in collaboration with community partners.	• Delivery system redesign • Support from the health care organization • Linkage to community resources	Qualitative study including 7 semi-structured interviews and 3 focus groups of 22 participants exploring the evolution of the IHH	Key to the development of the IHH was communication at all levels, an organic, flexible approach to program development, strong and committed leadership, staff engagement and support, and addressing competing priorities.
Zatloff et al. 2021 (98)	Primary care clinic in an outpatient behavioral health center serving adults with SMI in Atlanta, providing primary care services alongside psychiatric and behavioral healthcare.	Delivery system redesign	Retrospective chart review comparing medical outcomes and care utilization patterns for 147 patients the year prior and following introduction of the primary care clinic.	ED visits significantly decreased [*t*(146) = 3.98, *p* < 0.001] and Primary Care appointments significantly improved [*t*(136) = 14.50, *p* < 0.001] post implementation. Medical outcome changes (HbA1c, cholesterol, blood pressure, body mass index) were not significant.
Chambers et al. 2023 (69)	This PBHCI program established a full scope primary care clinic co-located with behavioral health services in Western New York. Medical directors from family medicine and psychiatry oversaw patient care. The program relied on physician assistants, nurses, case managers, peer workers, and specialized staff for coordination and whole-person care.	• Delivery system redesign • Support from the health care organization • Linkage to community resources	A retrospective chart review of 532 adults with SMI who completed at least two National Outcome Measures (NOMs) assessments over a four-year period (2015–2019).	Significant reductions were observed in the percentage of participants with blood pressure ≥120/80 mmHg (27.4% to 20.0%, *p* < 0.05) and total cholesterol ≥200 mg/dl (12.0%–8.3%, *p* < .05). Waist circumference and breath CO worsened significantly over the study period.
Eldridge et al. 2011 (105)	The Well-Being Support Program (WSP) was a four-session package delivered by trained mental health practitioners in South East England. Key program components included physical health screening, lifestyle interventions, referral to appropriate services and strengthening primary-secondary care links.	• Support of patient self-management • Delivery system redesign	Evaluation of the WSP over a year, leveraging program enrollment data and qualitative interviews with 6 providers.	Of the 754 enrolled patients, 159 (21%) completed the program. Mean change in BMI was not statistically significant. A significant improvement in blood pressure was observed in 17 patients (14%), while 18 patients (15%) showed worsening hypertension. Qualitative feedback was largely positive.
Siantz et al. 2016 (129)	Integrated behavioral health pilot programs including peer providers serving adults with SMI in Los Angeles County Department of Mental Health (DMH). The pilot programs included five co-located primary and behavioral health care programs, eleven partnerships coordinating care across different sites, and five Housing First ACT teams.	Delivery system redesign	Site visits, chart reviews, semi-structured interviews with providers and clinic observations evaluating the implementation of peer services in 24 integrated care programs.	15 of 24 programs included peer providers, with varying roles across program types. 10 of 14 integrated programs had infrastructure for training and supervision of peer providers. A culture of stigma influence use of peer providers.
Ma and Saw 2018 (102)	Three primary care providers integrated into a large community mental health clinic in California, serving low income Asian immigrants. Patients saw primary care physicians every three months, with bilingual care managers assisting.	• Support of patient self-management • Use of clinical information systems • Delivery system redesign • Support from the health care organization	Qualitative study including 5 semi-structured interviews with providers and seven focus groups with 41 patients, exploring facilitators and barriers to primary care-behavioral health integration in a multilingual setting.	Workforce limitations and payment structures hindered care integration. Improving organizational culture and practice, communication, and patient engagement facilitated successful implementation and improved outcomes.
Wells et al. 2019 (130)	Primary care practices in ten community mental health centers CMHCs) in Texas leveraging Medicaid 1115 waiver funding to integrated primary care into existing mental health services for adults with SMI: four CMHCs hired primary care providers, four partnered with federally qualified health centers, and two contracted with independent providers.	• Support of patient self-management • Use of clinical information systems • Delivery system redesign • Support from the health care organization	Case study including site visits to 10 CMHCs, 66 interviews with leadership and staff and focus groups with 75 patients as well as follow-up phone interviews with key staff informants one year later.	Findings highlight the importance of the scope of services provided on-site, and of communication and coordination between providers, as well as success in scaling up integration quickly despite challenges in provider and patient recruitment and retention. Patients reported positive experiences with integrated care.
Connor et al. 2018 (78)	Two levels of integrated physical health services in 22 behavioral health clinics in New York State: 1. Health Monitoring (HM), including regular assessment of health indicators (blood pressure, BMI, smoking, activity level). 2. HM plus Health Physicals (HM/HP), including HM plus annual comprehensive physical evaluations	Delivery system redesign	Cost analysis using data collected from interviews and financial reports from 14 clinics providing HM and 8 clinics providing HM/HP.	The mean annual budgets for HM and HM/HP clinics were $2.2M and $3.1M respectively. Direct costs for HPs were $67 per visit and for HM $18 per visit, with annual care coordination costs $66,700 in HM clinics compared to $67,200 in HM/HP clinics.
Ramanuj et al. 2018 (79)	11 behavioral health settings in New York City offering integrated care through the Primary and Behavioral Healthcare Integration (PBHCI) initiative and 3 Federally Qualified Health Centres (FQHCs) offering integrated care through PBHCI or the Delivery System Reform Incentive Program (DSRIP).	PBHCI: see Scharf, et al. 2014 (89).	Qualitative study engaging 36 senior clinicians and administrators in group interviews and 16 frontline staff in individual interviews exploring barriers and facilitators to integrated care.	Facilitators included teamwork, co-location of care, and care coordination. Barriers included regulatory fragmentation, licensing, and reimbursement mechanisms. Organizational culture and leadership were important mediators of integrated care.
Scharf et al. 2013 (81)	56 behavioral health grantees across 26 U.S. states funded through the Primary and Behavioral Health Integration Initiative (PBHCI) to establish: screening, assessment, and referral of adults with SMI for general medical illnesses and risk factors; a registry or tracking system for physical health needs/outcomes; care management; prevention and wellness support services.	• Support of patient self-management • Use of clinical information systems • Delivery system redesign • Support from the health care organization	Data were collected from grantee proposals, semi-structured interviews with core staff, and quarterly reports to assess early implementation experiences.	Grantees varied in size, location, and service integration approaches. Implementation barriers included space constraints, staff recruitment and retention, data management, and patient recruitment.
Breslau et al. 2021 (100)	Primary and behavioral Health Integration Initiative (PBHCI): see Scharf, et al. 2014 (89).	PBHCI: see Scharf et al. 2014 (89).	Medicaid claims data was used to estimate the impact of PBHCI grants on utilization, costs of care, and quality, using a difference-in-differences model to compare PBHCI grantee clinics with comparison clinics.	PBHCI successfully reduced frequent use of emergency and inpatient services for physical health conditions, lowered care costs, and improved follow-up after hospitalization for mental illness. The effect on quality of preventive care and health monitoring for chronic physical conditions was mixed.
Bandara et al. 2020 (101)	Maryland's Behavioral Health Home (BHH) program, implemented in psychiatric rehabilitation programs (PRPs) for adults with SMI focuses on six core areas: comprehensive care management, care coordination, health promotion, comprehensive transitional care and follow-up, individual and family support, and referrals to community and social support services.	• Support of patient self-management • Use of clinical information systems • Delivery system redesign • Support from the health care organization	Medicaid claims data for 12,232 individuals with SMI enrolled in a PRP (3,319 enrolled in BHH; 8,913 non enrolled in BHH) was examined to assess BHH impact on healthcare utilization.	BHH enrollment was associated with reduced probability of all-cause (PP: 0.23 BHH enrollment vs. 0.26 non enrollment: *p* < 0.01), and physical health ED visits (PP: 0.21 BHH enrollment vs. 0.24 non enrollment, *p* < 0.01) and no effect on inpatient admissions.
McGinty et al. 2020 (131)	Maryland Medicaid BHH, see Bandara et al. 2020 (101).	See Bandara et al. 2020 (101)	Retrospective cohort analysis using administrative data to compare quality of cardiovascular (CVD) care among adults with SMI and diabetes (914 BHH enrolled; 1,691 non- BHH enrolled) and CVD (601 BHH enrolled; 1,298 non-BHH enrolled) before and after BHH implementation.	BHH enrollment was associated with increased likelihood of receiving an annual eye exam for participants with diabetes (OR 1.86, 95% CI 1.19–2.91), but no changes in other care quality measures (e.g., HbA1c, diabetic nephropathy, and cholesterol testing).
Stone et al. 2020 (82)	Maryland Medicaid BHH, see Bandara et al. 2020 (101).	See Bandara et al. 2020 (101)	The study examines the Maryland's policy environment supporting BHH implementation using the policy ecology framework.	Existing policies fail to address key implementation barriers, including difficulties coordinating with external providers, inadequate health IT, lack of population health management capacity, staffing shortfalls, and consumer engagement issues.
Tatreau et al. 2016 (132)	A physician's assistant supervised by a family physician providing reverse collocated care (RCL) through daily coverage to an inpatient psychiatric unit serving adults with SMI in North Carolina, offering admission consultations, treating patients with comorbid medical conditions, and obtaining necessary lab values.	Delivery system redesign	Chart review to compare the screening and treatment of medical comorbidities among adults consecutively admitted to two psychiatric units and discharged on second generation antipsychotics; one unit (*n* = 220) offered RCL and the second (*n* = 232) offered treatment as usual (TAU).	The TAU group had significantly more screening lab tests including HbA**1c** tests (56% vs. 16%, *p* < 0.001), glucose (99% vs. 66%, *p* < 0.001), and lipids (61% vs. 20%, *p* < 0.001), but RCL group had higher responses to abnormal tests. Patients in the RCL group were more likely to be diagnosed with obesity, tobacco use disorder, and hyperlipidemia and receive treatment for hypertension and hyperlipidemia (76% vs. 58%, *p* < 0.001 for hypertension; 37% vs. 8%, *p* < .005 for hyperlipidemia).
Woltmann et al. 2024 (133)	Community mental health programs with behavioral health homes (BHHs) serving adults with SMI in Maryland and Michigan during the COVID pandemic. BHHs offer coordinated physical health care, health promotion, individual and family support, and referrals to community and social support services	• Delivery system redesign • Support of patient self-management • Use of clinical information systems • Support from the health care organization	Through interviews with among 72 providers across 21 sites, the study explored barriers and strategies for implementing and sustaining BHHs during the pandemic.	Patients struggled with access and effective use of digital platforms, while staff reported service disruptions, difficulties monitoring vital signs remotely and maintaining strong collaboration with primary care providers. Additionally, participants perceived virtual encounters as less effective than in-person sessions.
Flanagan et al. 2024 (103)	Co-location of a primary care center (PCC) and a community mental health center (MHC) in a mid-sized city in northeaster US serving adults with SMI; PCC and MHC providers have separate systems, communicate via phone/email about shared patients and meet only as needed.	• Delivery system redesign • Support from the health care organization	Five focus groups with 48 participants (providers, administrators, patients), and 2 online surveys examined the integration of PCC and MHC services. 50 participants responded to the first online survey and 41 to the second survey.	There was limited staff awareness of the PCP in the first year and co-location alone was not sufficient to promote integrated care. There was a desire to strengthen integration via shared medical records and enhanced communication between providers.
Burner et al. 2024 (134)	A certified community behavioral health clinic (CCBHC) in Midwestern US incorporating primary care into their outpatient clinic to offer integrated services to adults with SMI in a Midwestern US community via close onsite collaboration and shared systems (e.g., scheduling and medical records)	• Delivery system redesign • Use of clinical information systemsLinkage to community resources	Using qualitative interviews and surveys with 40 patients and 5 providers, the study assessed patient and provider needs, satisfaction, and level of care integration.	Patients using integrated primary care reported higher satisfaction (average score 4.9, SD 0.32) and stronger intent to keep seeing their providers (score 5.0, SD 0.0) compared to those in non-integrated care. Co-location alone, without effective communication and practice changes, was insufficient to achieve true integration.
Utter et al. 2023 (135)	Integrated care in outpatient mental health treatment facilities in the US, typically involving a multidisciplinary team developing a single treatment plan, collectively monitoring patient progress, and coordinating care through shared payment and billing structures.	• Delivery system redesign • Support from the health care organization	A secondary analysis of publicly available facility level data (*n* = 9,889) to assess the integration of primary care services in specialty outpatient mental health facilities over time.	While integrated care increased modestly from 2015 to 2020, overall prevalence remains low at 17.5% of facilities. Access to integrated services varies by state, with several regions showing declining availability.
Kogan et al. 2017 (106)	One of two distinct care approaches implemented across 11 community mental health providers in Pennsylvania serving adults with SMI: 1. Patient Self-Directed care: used a secure web portal to offer patients support, education and resources in learning about their health and taking an active role in their care 2. Provider-Supported care: involved registered nurses supporting patients with care coordination, provider communications, and wellness supports and resources	• Support of patient self-management • Delivery system redesign • Support from the health care organization • Linkage to community resources	Cluster-randomized trial recruiting Provider-Supported (*n* = 713) and Patient Self-Directed Care participants (*n* = 516) across 11 provider sites.	Challenges in conducting the trial included intervention training and implementation challenges, participant recruitment and retention, and data collection challenges
Nikolajski et al. 2022 (104)	See Kogan et al. 2017 (106)	See Kogan et al. 2017 (106)	65 interviews with 30 staff exploring perceptions of barriers and facilitators to BHH implementation at baseline, and 1 and 2 years following implementation.	Staff turnover, hesitation to change, and competing acute client needs were major barriers to implementation. Agency-wide culture shifts toward wellness, strong leadership support, and integration into daily workflows were critical success factors.
Schuster et al. 2018 (94)	See Kogan et al. 2017 (106)	See Kogan et al. 2017 (106)	See Kogan et al. 2017 (106)	Both approaches increased patient activation in care (more rapid increase for provider supported participants). Health status and engagement in primary and specialty care increased in both groups, with no between-group differences.

ACT, assertive community treatment; BH, behavioral health; BHH, behavioral health home; BMI, body mass index; CAMH, Centre for Addiction and Mental Health; CMHA-D, Canadian Mental Health Association-Durham; CMHC, Community Mental Health Center; CQUIN, Commissioning for Quality and Innovation; DSRIP, Delivery System Reform Incentive Program; ECG, Electrocardiogram; ED, Emergency Department; EHR, Electronic Health Record; ER, Emergency Room; FQHC, Federally Qualified Health Center; GP, General Practitioner; HIP, Health Improvement Profile; HOPES, Helping Older People Experience Success; IT, Information Technology; NHS, National Health Service; NP, Nurse Practitioner; NYS OMH, New York State Office of Mental Health; PBHCI, Primary and Behavioral Health Care Integration; PCARE, Primary Care Access Referral and Evaluation; PRP, Psychiatric Rehabilitation Program; RCL, Reverse Colocation; SAMHSA, Substance Abuse and Mental Health Services Administration; SMI, Severe Mental Illness; TAU, Treatment As Usual.

A randomized controlled trial (RCT) by Druss et al. (92) found that integrated care participants received significantly more preventive services and had significantly lower Framingham cardiovascular risk scores compared to controls at 12 months (6.9% vs. 9.8%, *p* = 0.02). In a more recent RCT, Errichetti et al. (93) demonstrated that intervention group participants had significantly lower systolic blood pressure (adjusted mean difference −3.86, *p* = 0.04) and average Hemoglobin A1C (HbA1c; adjusted mean difference −0.36, *p* = 0.001) compared to controls at 12 months, with no differences found in diastolic blood pressure, body mass index (BMI), or cholesterol. Other RCTs demonstrated that integrated care models can improve processes and quality of care (e.g., screening rates for preventive care) if not health and wellness outcomes (49, 55, 68, 84, 94).

In a quasi-experimental study, Scharf et al. (95) reported that compared to control clinic consumers, PBHCI consumers showed greater mean reductions in total and low-density lipoprotein (LDL) cholesterol (36 mg/dl, *p* < 0.01 and 35 mg/dl *p* < 0.001 respectively), and greater mean increases in high-density lipoprotein (HDL) cholesterol (3 mg/dl, *p* < 0.05), though no significant effects were observed for other health indicators such as BMI and HbA1c.

In a controlled retrospective cohort study, Iturralde et al. (77) showed that participants of a pharmacist-led collaborative care model for adults with SMI, compared to controls, achieved greater glycemic (ARD = 9.3; 95%CI = 5.0–13.7) and lipid screening (ARD = 13.0; 95% CI = 8.1–17.9) and increased EKG evaluations (ARD = 6.8; 95% CI = 2.0–11.5) from pre- to post-enrollment compared to propensity matched control participants.

In other studies, Gilmer et al. (96) found that highly integrated programs, compared to programs with low integration levels, led to greater improvements in physical health status (*p* < 0.01), higher screening rates for blood pressure, cholesterol and blood glucose (all at *p* < 0.01), a decline in the number of patients who were identified with hypertension and an increase among those identified with prediabetes or diabetes (both at *p* = 0.01). Similarly, Johnson et al. (70) found that Behavioral Health Home (BHH) participation, compared to no participation, was associated with 0.29 fewer percentage points for HbA1c (*p* < 0.05) with no changes noted in LDL cholesterol.

Non-controlled studies also reported on the effects of integrating care on common health indicators. A longitudinal cohort study by Pirraglia et al. (97) found that a primary care clinic co-located in a mental health setting for veterans with SMI had significantly improved goal attainment for blood pressure (adjusted odds ratio [AOR] = 2.16; 95% confidence interval [CI], 1.47–3.18), LDL cholesterol (AOR = 1.60; 95% CI, 1.10–2.34), triglyceride (AOR = 1.64;95% CI, 1.06–2.51), and BMI (AOR = 1.81; 95% CI, 1.29–2.54), though changes in HDL cholesterol and HbA1c were not significant. In a pre-post retrospective chart review, Chambers et al. (69) reported a decrease in the percentage of participants with a blood pressure over ≥120/80 mmHg (27.4% vs. 20.0%, *p* < 0.05) and ≥200 mg/dl total cholesterol (12.0% vs. 8.3%, *p* < 0.05) between 2015 and 2019, though worsened outcomes were observed in waist circumference during the same period.

### Health service use outcomes

Eleven studies reported on healthcare utilization outcomes, with select findings described below (see Table 2 for study details). Integrating physical health care into mental health settings was found to have generally positive impacts on healthcare utilization. Primary care and general medical outpatient care access showed improvements in most studies, while emergency and inpatient service use demonstrated promising but not uniformly positive results.

Johnson et al. (70) reported that BHH enrollees experienced an immediate increase in primary care visits, with 0.18 more visits per month compared to non-BHH participants (*p* < 0.01). They also reported an increase in general medical outpatient visits per month compared to non-BHH participants (+0.055, *p* < 0.01). Similarly, Krupski et al. (85) reported that a higher proportion of PBHCI program enrollees in Washington State used outpatient medical services at two sites following program enrollment, compared to propensity matched controls from the same sites. Specifically, the percentage of PBHCI enrollees using outpatient medical services increased from 80% to 92% in site 1, and from 39% to 76% in site 2, compared to limited changes in the control groups (*p* < 0.003 and *p* < 0.001 respectively). In contrast, Breslau, Leckman-Westin, Yu, et al. (98), in a quasi-experimental study using administrative health data observed no differences on the odds of having an outpatient medical visit between PBHCI enrollees and control participants in New York State. Similarly, Iturralde et al. (77) found no differences in primary care visits between participants of a pharmacist led collaborative care model and control participants in northern California.

In non-controlled studies, Pirraglia et al. (97) reported that enrollment in a collocated primary care clinic was associated with a significant increase in primary care visits among veterans with SMI and poor primary care engagement, with the median number of visits increasing from 0 to 2 post-implementation (*p* < 0.001). More recently, Zatloff et al. (98), using a pre-post retrospective chart review, reported significant improvements in the percentage of primary care appointments attended over a one-year period after integrating primary care services within an outpatient behavioral health clinic [*t*(136) = 14.50, *p* < 0.001].

Emergency department (ED) use and hospitalization patterns revealed more complex outcomes across studies. Bartels et al. (68), in a randomized controlled trial, found no change in acute service use at the three-year follow-up of a preventive healthcare intervention for older adults with SMI. Breslau, Leckman-Westin, Han, et al. (99), using Medicaid claims data, found that hospital stays for medical conditions increased significantly in PBHCI clinics in New York City compared to control clinics, possibly due to these programs uncovering previously unidentified physical health needs. The relative odds of hospitalization for a medical diagnosis in PBHCI vs. control clinics was 1.21 (95% CI: 1.10–1.32) in wave 1 and 1.33 (95% CI: 1.07–1.65) in wave 2 of PBHCI grant implementation. Furthermore, there was no significant association between PBHCI enrollment and the likelihood of having an ED visit with a medical diagnosis.

In another PBHCI study, Krupski et al. (85) found that program enrollment was associated with a reduction in the proportion of enrollees with inpatient admissions (from 18% to 12%) at one of two sites, compared to propensity-matched control participants (a reduction from 15% to 17%; *p* < 0.04) but had no significant impact on emergency department use at either site. Using Medicaid claims data from three States, Breslau et al. (100) found that PBHCI program implementation was associated with a reduction in the proportion of enrollees having four or more ED or inpatient visits compared to control clinics, with statistically significant decreases observed in three of the five PBCHI cohorts examined. The reduction in frequent utilization was specific to health service utilization for physical health conditions.

Bandara et al. (101) on the other hand reported that Maryland's BHH program was associated with a reduction in the odds of having an all-cause ED visit compared to non-enrollment (OR:0.87, *p* < 0.01), though there was no effect on inpatient admission rates per person in a three-month period. The reduction in ED utilization was driven by a reduction in the predicted probability (PP) of having a physical health ED visit in a 3-month period among BHH enrollees (PP: 0.21 BHH enrollment vs. 0.24 non-enrollment, *p* < 0.01). Similarly Tepper et al. (91) reported that the total number of ED visits per capita decreased significantly among BHH enrollees compared with control participants (*p* = 0.014). Total psychiatric hospitalizations per capita similarly declined for BHH patients in that study, but remained stable for control group participants (*p* = 0.002). There were no differences in either the rate or the number of general medical hospitalizations. Furthermore, Johnson et al. (70) reported that relative to control group participants, BHH enrollees had an immediate decrease in emergency department visits (–0.031 visits/month, *p* < 0.01). They also reported that while inpatient visits decreased over time for both BHH enrollees and control participants before BHH implementation, they decreased more slowly for BHH patients post-implementation. More recently, Iturralde et al. (77) found no significant differences in ED use and hospitalizations between participants of a pharmacist led collaborative care model for adults with SMI and control participants. Lastly, Goh et al. (75), in a retrospective file audit, analyzed admissions to assess medical comorbidities and interventions, finding that adding a medical resident to an inpatient psychogeriatric unit did not affect emergency medical transfer rates.

### Barriers and facilitators of implementation

Seventeen studies discussed barriers and facilitators to implementing integrated care initiatives in mental health settings. Multidisciplinary teams, care coordination, administrative support and organizational cultures emphasizing shared responsibility and collaboration were found to facilitate implementation (47, 82, 90, 102). Furthermore, improved organizational communication and patient engagement were associated with enhanced participant outcomes (102, 103).

Finally, effective teamwork, characterized by clearly defined roles and responsibilities among team members, attention to daily workflows, and connection to community programs were found to be crucial for effective collaboration among providers (79, 82, 88, 89, 104). A clear vision emphasizing integration of physical and mental health care in the organization's mission, and leveraging data systems, were also highlighted as essential, along with strong leadership, aligning efforts and resources (79, 86, 102).

The most frequently identified challenges of implementation include securing adequate financial resources, usability and maintenance of clinical information systems, population health management capacity, lack of care coordination, staff retention, and patient enrollment (47, 79, 81, 82, 89, 90, 103–109). Time-limited funding was identified as an ongoing challenge across different settings (79, 81).

McGinty et al. (108), Daumit et al. (47) and Stone et al. (82) reported that Medicaid Behavioral Health Homes (BHH) in Maryland faced tensions between population health management and direct clinical care, and implementation barriers related to limited staff experience, health information technology usability, difficulty engaging external service providers and state regulations impacting service delivery. Workforce limitations, such as high client-to-staff ratios and frequent staff turnover, further complicated care delivery (47, 82).

Scharf et al. (89) highlighted that across 3 integrated care initiatives in New York State, implementation barriers included licensing requirements, information sharing between providers, infrastructure, and sustainability challenges. In other settings, payment structures and low wages for community mental health work were noted to exacerbate staff retention issues (102, 107).

Engaging primary care providers (PCPs) remained a significant challenge in several settings. Negative attitudes toward patients with SMI and limited incentives contributed to low PCP participation in care coordination (47, 107).

Despite these challenges, programs like PBHCI, and Maryland's BHH demonstrated that with robust funding, strong leadership, and effective communication strategies, integrated care models could reduce costs and improve outcomes when tailored to local needs and supported by multi- disciplinary collaboration (89, 100).

### Costs and financing

Six studies explored the costs and savings associated with integrating care in behavioral health settings, focusing on funding and reimbursement strategies, cost-savings and sustainability.

In the initiatives examined, funding was allocated through various mechanisms. Ramanuj et al. (79) reported that the PBHCI program, administered by the Substance Abuse and Mental Health Services Administration (SAMHSA), provided $400,000 per year for four years to enable behavioral health clinics to offer primary care services. The program, initiated in 2009, had awarded 189 grants by 2015, with an average of 250 enrollees per grantee. In 2010, in complementary efforts, the New York State Office of Mental Health introduced regulations designed to promote physical health care in mental health clinics by allowing partial reimbursement for health monitoring and health physicals through Medicaid, although insufficient reimbursement for high-cost services hindered adoption (78). The same year, the Affordable Care Act Medicaid health home waiver allowed states to create Medicaid health homes, including behavioral health homes, to provide care coordination and health promotion services for beneficiaries with complex health needs (101). Regarding sustainability, the temporary nature of PBHCI grants was a noted barrier, as clinics struggled with fragmented funding. Ramanuj et al. (79) further concluded that sustaining integration efforts required investments in infrastructure, such as electronic health records and care quality monitoring.

Integrated care models achieved variable outcomes in terms of cost savings. An assessment of a medical care management intervention in community mental health settings serving adults with SMI found a $932 reduction per patient in total costs by the second year of the intervention, with a 92.3% probability of being associated with lower costs than usual care [95% CI (−1973, 102)] (48). The study also highlighted that community mental health centers would need at least 58% of their patients to have Medicaid or other insurance for the program to break even financially. Since only 40.5% of enrollees had Medicaid at the study site, the program appeared unsustainable in the long term (48).

Krupski et al. (85) comparing PBHCI clients with propensity matched controls at 2 sites, found that PBHCI participation was associated with a trend toward reduced inpatient hospital costs per participant per month at one site (–$217.68, *p* = .06), although no hospital-related cost savings were observed at a second site. Breslau et al. (101), evaluating PBHCI outcomes across three states, found that PBHCI participation was associated with a reduction in the total costs of care per consumer in three of the five cohorts examined, and no significant cost differences in the remaining two cohorts, compared to control sites. Further, sources of cost reduction varied across cohorts: outpatient costs decreased in two cohorts, while emergency department-related costs showed mixed results, increasing in one cohort and decreasing in another.

Connor et al. (78) examined the financial impact of providing physical health monitoring or physical health monitoring plus health physicals for adults with SMI in specialty mental health clinics in New York State, highlighting significant cost barriers. Health physicals were estimated to cost $153 on average but were reimbursed at lower Medicaid rates ($89.48–$129.28). Similarly, health monitoring sessions cost $51, while reimbursements ranged from $33.79 to $48.82. Additional costs for care coordination, such as referrals and follow-ups, strained clinic budgets, especially for freestanding facilities. The authors highlighted these gaps as barriers to sustainability and widespread adoption and called for policies to address them.

## Discussion

With a growing interest in addressing the mortality gap among adults living with SMI internationally, a variety of policy initiatives and integrated care models have been described in the literature in recent years. This scoping review sought to examine integrated service delivery models and clinical practices within mental health settings serving adults with SMI and their outcomes, aiming to capture service delivery and practice innovations in this important area.

Most integrated service delivery models and clinical practices described in this review were implemented in community mental health settings in the United States. The service delivery models examined, although often not described in detail, typically involved collocated or integrated primary care professionals, and generally leveraged several components of Wagner's Chronic Care Model, emphasizing delivery system redesign, patient self-management support, and use of clinical information systems. Funding and leadership support, effective teamwork, care coordination, and leveraging data systems were central to implementation efforts (79, 89, 102). Several implementation challenges were highlighted by stakeholders. These included reimbursement mechanisms, high staff turnover rates, difficulties in engaging primary care providers to treat people with SMI, and communication and coordination between team members (47, 78). Furthermore, challenges with poor health information system usability, were common (108). Overall, integrated care models were noted to require investments in comprehensive workforce training, continuous improvement of clinical information systems, and sustained implementation support. Longitudinal evaluation and dynamic adaptation of these models, informed by implementation science tools, such as the Consolidated Framework for Implementation Research, will be helpful to ensure they meet the evolving needs of both patients and healthcare providers (110).

Physical health indicator and healthcare utilization outcomes showed promising results. Inconsistent improvements across health outcomes are not uncommon in the early stages of implementing service delivery changes (95), and program implementation challenges and limited patient engagement may have reduced program effectiveness in some studies. Although some of the mixed outcomes may reflect the need for service improvements (111), managing complex conditions such as diabetes and obesity necessitates both medical interventions and significant behavioral changes, which can be more challenging to achieve and sustain and generally require longer-term follow up to see improvements (112, 113). Notably, significant improvements were observed in cholesterol and blood pressure in some programs (69, 89, 95–97). As approximately 44% of the decrease of death from coronary heart disease in the general population has been attributed to changes in risk factors, including reductions in total cholesterol and systolic blood pressure (114), these improvements underscore the achievements of the integrated care models, and their potential to improve health outcomes and life expectancy in this population.

While most integrated care models increased primary care visits, there were inconsistent impacts on emergency department visits and hospitalizations, highlighting that additional attention is needed to the complex care needs of this population (70, 85, 98, 99). On the other hand, the observed increases in medical hospitalizations in some programs, particularly during early implementation, may represent a positive outcome by identifying and addressing previously unmet medical needs in this historically underserved population. This understanding emphasizes the importance of considering the complex pathway to improving physical health outcomes among adults living with SMI, and the need for tailored person-centered interventions. Short-term follow-up times may also contribute to the lack of integrated care impact on general medical inpatient utilization, as intervention components may require longer timelines to effect positive change (91).

Strengths of this review include the use of rigorous methods and the assistance of a health librarian. Furthermore, the review examined service delivery models and clinical practices that addressed health needs comprehensively rather than focusing on a single disease or on individual risk factors. This approach renders findings more relevant for service planning and policy development aimed at improving general health. Finally, the review was enriched by input from a diverse study team, inclusive of individuals living with SMI and family members, health services researchers, and clinicians serving this population. The inclusion of these varied perspectives guided our efforts to ensure our work, including our research questions and synthesis and interpretation of key findings, is relevant to key stakeholders. As this scoping review focuses on the breadth of research rather than the quality, it does not include a quality assessment of the included articles. The heterogeneity of the interventions further complicates this issue, making it difficult to apply a standardized quality assessment across all sources. Further, the vast differences in health system structures, organization and funding models across countries and jurisdictions highlights the need for caution in interpreting findings, as interventions that are successful, feasible and acceptable in one context may not be applicable or necessarily yield the same effectiveness or cost outcomes in another. Finally, this scoping review was limited to the academic literature published since 2010, potentially missing models of care or practices captured in the grey literature or earlier academic publications. To address the mortality gap among adults living with SMI, health systems and policy makers need to address all contributing factors, beyond healthcare delivery, including substance use, medication side effects and the social determinants of health affecting this population. Despite its limitations, this review offers important insights into opportunities to advance integrated physical and mental health care delivery for people with SMI within diverse mental health settings, setting the stage for more comprehensive policy interventions.

Future research should continue to examine the effectiveness of integrated care interventions over longer periods of time, to assess long term effectiveness. Further, future studies should offer more detailed program descriptions and include measures of engagement among adults with SMI, as current service delivery models continue to present barriers to engagement (115). This understanding will allow for the refinement and dynamic adaptation of service innovations, and improve their acceptability to service users. There is a notable gap in the literature on aging with SMI. While our review included studies on older adults, the limited research available highlights the need for further exploration in this area, given the challenges of cognitive comorbidities and accelerated aging in this population (116, 117). Finally, no studies addressed the physical health needs of long-term psychiatric inpatients, such as forensic inpatients, which may be difficult to address outside of general hospital settings. These long-term inpatient psychiatric care models require attention to improve the quality and comprehensiveness of care and reduce rates of emergency medical transfers to general hospital settings (75).

In conclusion, this scoping review examined service delivery models and clinical practices aimed at integrating physical health care within mental health settings for adults living with SMI. Although studies of integrated care models demonstrated improvements in some physical health indicators and aspects of health care utilization, further efforts are needed to achieve sustained improvements in a range of health domains and ultimately, reduce health disparities in this population. These findings underscore the necessity of ongoing efforts to address the health needs of this population comprehensively and of evaluating the effectiveness of these interventions over time.

## Data Availability

The original contributions presented in the study are included in the article/Supplementary Material, further inquiries can be directed to the corresponding author.
