# Supplementary material for: Strengthening the delivery of integrated physical health care for adults experiencing serious mental illness: a scoping review of interventions in mental health settings
Source: Front Health Serv. 2025 Jun 20;5:1570100. doi: 10.3389/frhs.2025.1570100 (PMC12226505; doi:10.3389/frhs.2025.1570100)
Supplement: Supplementary file 1 [file Table1.docx]

| **SEARCH STRATEGY:**  Ovid MEDLINE: Epub Ahead of Print, In-Process & Other Non-Indexed Citations, Ovid MEDLINE® Daily and Ovid MEDLINE® <1946-Present> | |
| --- | --- |
|  |  |
| 1 | exp Primary Health Care/ |
| 2 | Chronic disease/ |
| 3 | primary health*.ti,kf. |
| 4 | physical health.ti,kf. |
| 5 | physical condition*.ti,kf. |
| 6 | somatic condition*.ti,kf. |
| 7 | primary medic*.ti,kf. |
| 8 | primary care.ti,kf. |
| 9 | (chronic disease* or chronic illness*).ti,kf. |
| 10 | ((cardiovascular or metaboli* or diabet* or lifestyle* or life style* or (cancer adj2 screen*)) adj3 (service* or treatment* or therap* or intervention* or program*)).ti,ab,kf. |
| 11 | (primary health* or physical health or physical condition* or somatic condition* or primary medic* or primary care or chronic disease* or chronic illness*).ab. /freq=3 |
| 12 | or/1-11 |
| 13 | ((integrat* or implement* or coordinat* or co-ordinat* or co-locat* or colocat* or embed* or partnership* or combin*) adj5 ((psychiatr* or psycholog* or mental* or addict* or "substance use" or alcohol* or "use disorder*" or mood or affective or depress* or anxiety or personality disorder* or psychosis or psychoses or psychotic or schizo* or obsessive or ocd or ptsd or posttraumatic or suicid* of self-harm* or selfharm*) adj3 (service* or treatment* or intervention* or program* or setting* or environment* or facility or facilities or institut* or clinic or clinics or centre* or center* or unit or units or ward or wards))).ti,ab,kf,hw. |
| 14 | ((integrat* or implement* or coordinat* or co-ordinat* or co-locat* or colocat* or embed* or partnership* or combin*) adj5 ((psychiatr* or mental*) adj2 (client* or patient* or inpatient* or outpatient* or service user*))).ti,ab,kf,hw. |
| 15 | ((integrat* or implement* or coordinat* or co-ordinat* or co-locat* or colocat* or embed* or partnership* or combin*) adj5 (support* housing or support* accommodation or "housing first")).ti,ab,kf,hw. |
| 16 | ((integrat* or implement* or coordinat* or co-ordinat* or co-locat* or colocat* or embed* or partnership* or combin*) adj5 ((severe or persisten* or serious*) adj1 (mental* or psychiatr*))).ti,ab,kf,hw. |
| 17 | or/13-16 |
| 18 | 12 and 17 [PC + integrated MH words] |
| 19 | Mental Health Services/ |
| 20 | Community Mental Health Services/ |
| 21 | Emergency Services, Psychiatric/ |
| 22 | Mentally Ill Persons/ |
| 23 | Drug Users/ |
| 24 | Hospitals, Psychiatric/ |
| 25 | exp Psychiatry/ |
| 26 | ((psychiatr* or psycholog* or mental* or addict* or "substance use" or alcohol* or "use disorder*" or mood or affective or depress* or anxiety or personality disorder* or psychosis or psychoses or psychotic or schizo* or obsessive or ocd or ptsd or posttraumatic or suicid* of self-harm* or selfharm*) adj3 (service* or treatment* or intervention* or setting* or environment* or facility or facilities or institut* or clinic or clinics or centre* or center* or unit or units or ward or wards)).ti,ab,kf. |
| 27 | ((psychiatr* or mental*) adj2 (client* or patient* or inpatient* or outpatient* or service user*)).ti,ab,kf. |
| 28 | (support* housing or support* accommodation or "housing first").ti,ab,kf. |
| 29 | ((severe or persisten* or serious*) adj1 (mental* or psychiatr*)).ti,ab,kf. |
| 30 | or/19-29 |
| 31 | ((integrat* or implement* or coordinat* or co-ordinat* or co-locat* or colocat* or embed* or partnership* or combin*) adj5 (primary health* or physical health or physical condition* or somatic condition* or primary medic* or primary care or chronic disease* or chronic illness* or cardiovascular or metaboli* or diabet* or (cancer adj2 screen*) or lifestyle* or life style*)).ti,ab,kf,hw. |
| 32 | 30 and 31 [MH + integrated PC words] |
| 33 | Delivery of Health Care/ |
| 34 | Delivery of Health Care, Integrated/ |
| 35 | implementation science/ |
| 36 | (integrat* or implement* or coordinat* or co-ordinat* or co-locat* or colocat* or embed* or combin* or partnership*).ti,kf. |
| 37 | ((integrat* or coordinat* or co-ordinat* or co-locat* or colocat* or embed* or combin* or partnership*) adj3 (mental* or behavio* or psychiatr* or psycholog* or psychotherapy*)).ab. |
| 38 | ((integrat* or coordinat* or co-ordinat* or co-locat* or colocat* or embed* or combin* or partnership*) adj3 (health or healthcare or care or service* or treatment*)).ab. |
| 39 | ((integrat* or coordinat* or co-ordinat* or co-locat* or colocat* or embed* or combin* or partnership*) adj3 (primary* or physical)).ab. |
| 40 | ((integrat* or implement* or coordinat* or co-ordinat* or co-locat* or colocat* or embed* or combin* partnership*) adj3 (model* or program)).ab. |
| 41 | (implement* adj3 (science* or strateg* or integrat* or coordinat* or co-ordinat* or co-locat* or colocat* or embed* or combin* or partner*)).ab. |
| 42 | (reverse* adj3 integrat*).ti,ab,kf. |
| 43 | or/33-42 [Integration concept] |
| 44 | 1 or 2 |
| 45 | 19 or 20 or 21 or 22 or 24 or 25 or mental disorders/ |
| 46 | 43 and 44 and 45 [integration + PC subject headings + MH subject headings] |
| 47 | 18 or 32 or 46 |
| 48 | limit 47 to yr="2000 -Current" |
